# Effectiveness of Electronic Reminders to Improve Medication Adherence in Tuberculosis Patients: A Cluster-Randomised Trial

**DOI:** 10.1371/journal.pmed.1001876

**Published:** 2015-09-15

**Authors:** Xiaoqiu Liu, James J. Lewis, Hui Zhang, Wei Lu, Shun Zhang, Guilan Zheng, Liqiong Bai, Jun Li, Xue Li, Hongguang Chen, Mingming Liu, Rong Chen, Junying Chi, Jian Lu, Shitong Huan, Shiming Cheng, Lixia Wang, Shiwen Jiang, Daniel P. Chin, Katherine L. Fielding

**Affiliations:** 1 National Center for Tuberculosis Control and Prevention, Chinese Center for Disease Control and Prevention, Beijing, China; 2 MRC Tropical Epidemiology Group, London School of Hygiene & Tropical Medicine, London, United Kingdom; 3 Jiangsu Province Center for Disease Control and Prevention, Nanjing, Jiangsu, China; 4 Chongqing Provincial Tuberculosis Dispensary, Chongqing, Chongqing, China; 5 Heilongjiang Provincial Tuberculosis Dispensary, Harbin, Heilongjiang, China; 6 Hunan Provincial Tuberculosis Dispensary, Changsha, Hunan, China; 7 Dafeng County Center for Disease Control and Prevention, Dafeng, Jiangsu, China; 8 China Office, Bill & Melinda Gates Foundation, Beijing, China; University of California, San Francisco, UNITED STATES

## Abstract

**Background:**

Mobile text messaging and medication monitors (medication monitor boxes) have the potential to improve adherence to tuberculosis (TB) treatment and reduce the need for directly observed treatment (DOT), but to our knowledge they have not been properly evaluated in TB patients. We assessed the effectiveness of text messaging and medication monitors to improve medication adherence in TB patients.

**Methods and Findings:**

In a pragmatic cluster-randomised trial, 36 districts/counties (each with at least 300 active pulmonary TB patients registered in 2009) within the provinces of Heilongjiang, Jiangsu, Hunan, and Chongqing, China, were randomised using stratification and restriction to one of four case-management approaches in which patients received reminders via text messages, a medication monitor, combined, or neither (control). Patients in the intervention arms received reminders to take their drugs and reminders for monthly follow-up visits, and the managing doctor was recommended to switch patients with adherence problems to more intensive management or DOT. In all arms, patients took medications out of a medication monitor box, which recorded when the box was opened, but the box gave reminders only in the medication monitor and combined arms. Patients were followed up for 6 mo. The primary endpoint was the percentage of patient-months on TB treatment where at least 20% of doses were missed as measured by pill count and failure to open the medication monitor box. Secondary endpoints included additional adherence and standard treatment outcome measures. Interventions were not masked to study staff and patients. From 1 June 2011 to 7 March 2012, 4,292 new pulmonary TB patients were enrolled across the 36 clusters. A total of 119 patients (by arm: 33 control, 33 text messaging, 23 medication monitor, 30 combined) withdrew from the study in the first month because they were reassessed as not having TB by their managing doctor (61 patients) or were switched to a different treatment model because of hospitalisation or travel (58 patients), leaving 4,173 TB patients (by arm: 1,104 control, 1,008 text messaging, 997 medication monitor, 1,064 combined). The cluster geometric mean of the percentage of patient-months on TB treatment where at least 20% of doses were missed was 29.9% in the control arm; in comparison, this percentage was 27.3% in the text messaging arm (adjusted mean ratio [aMR] 0.94, 95% CI 0.71, 1.24), 17.0% in the medication monitor arm (aMR 0.58, 95% CI 0.42, 0.79), and 13.9% in the combined arm (aMR 0.49, 95% CI 0.27, 0.88). Patient loss to follow-up was lower in the text messaging arm than the control arm (aMR 0.42, 95% CI 0.18–0.98). Equipment malfunction or operation error was reported in all study arms. Analyses separating patients with and without medication monitor problems did not change the results. Initiation of intensive management was underutilised.

**Conclusions:**

This study is the first to our knowledge to utilise a randomised trial design to demonstrate the effectiveness of a medication monitor to improve medication adherence in TB patients. Reminders from medication monitors improved medication adherence in TB patients, but text messaging reminders did not. In a setting such as China where universal use of DOT is not feasible, innovative approaches to support patients in adhering to TB treatment, such as this, are needed.

**Trial Registration:**

Current Controlled Trials, ISRCTN46846388

## Abstract 2

In a cluster-randomized controlled trial, Katherine Fielding and colleagues examine the effectiveness of electronic reminders for improving tuberculosis medication adherence.

## Editors' Summary

**Background:**

Tuberculosis—a contagious bacterial disease that usually infects the lungs—is a major global public health problem. Every year, about 9 million people develop tuberculosis and at least 1.3 million people die as a result. *Mycobacterium tuberculosis*, the organism that causes tuberculosis, is spread in airborne droplets when people with tuberculosis cough or sneeze. The symptoms of tuberculosis include cough, weight loss, and fever. Diagnostic tests for tuberculosis include sputum smear microscopy (microscopic analysis of mucus coughed up from the lungs), the growth of *M*. *tuberculosis* from sputum samples, and chest X-rays. Tuberculosis can be cured by taking antibiotics daily for several months (usually isoniazid, rifampicin, ethambutol, and pyrazinamide for two months followed by isoniazid and rifampicin for a further four months), but the emergence of multidrug-resistant *M*. *tuberculosis* is making tuberculosis increasingly hard to treat.

**Why Was This Study Done?:**

Because tuberculosis treatment is long and unpleasant, patients often fail to take all their drugs. To improve medication adherence, the World Health Organization recommends that health care workers supervise patients while they take their medication (directly observed treatment, DOT). However, DOT can be hard to implement. In China, for example, where 11% of tuberculosis cases occur, DOT cannot be implemented in many parts of the country, and the national tuberculosis control policy permits self-administered treatment and treatment monitored by family members. It is estimated that over half of individuals with tuberculosis in China self-administer their treatment, but, in 2010, 20% of patients treated using nationally recommended case-management approaches were lost to follow-up or failed to take their medications regularly. In this pragmatic cluster-randomized trial, the researchers investigate whether reminders delivered by mobile phone or by medication monitor boxes (which provide audio reminders to patients and record when the box is opened) might improve tuberculosis medication adherence in China. A pragmatic trial asks whether an intervention works under real-life conditions; a cluster-randomized trial randomly assigns groups of people (here, people living in different counties/districts) to receive alternative interventions and compares outcomes in the differently treated “clusters.”

**What Did the Researchers Do and Find?:**

The researchers assigned people newly diagnosed with tuberculosis in counties/districts to receive reminders about taking their antibiotics and about monthly follow-up visits via text messaging, a medication monitor, or both text messaging and a medication monitor (the intervention arms), or to receive standard nationally recommended care without electronic reminders (the control arm). All the trial participants (about 1,000 per arm) took their drugs out of a medication monitor box, but the box’s audio reminder function was switched off in the text messaging only and control arms. In the intervention arms, doctors were advised to switch participants with poor medication adherence (evaluated at follow-up visits) to either more intensive management or DOT, depending on the level of missed treatment doses. Compared to the control arm, the percentage of patient-months with at least 20% of the drug doses missed (called “poor adherence” and measured by pill counts and data from the medication monitor) was not significantly reduced in the text messaging arm, whereas poor adherence was significantly reduced by 42% and 51% in the medication monitor and the combined arms, respectively (a significant reduction is unlikely to have happened by chance). Notably, fewer patients were switched to intensive management or DOT than expected based on medication adherence evaluations.

**What Do These Findings Mean?:**

These findings show that, in China, the use of an electronic medication monitor box to remind patients to take their anti-tuberculosis drugs improved medication adherence. Interestingly, text messaging alone, which has been shown to improve adherence to antiretroviral therapy among HIV-positive individuals, did not improve medication adherence among patients with tuberculosis, possibly because the messages were too frequent or too impersonal, although this intervention (but none of the others) did reduce patient loss to follow-up. Battery problems with the medication monitor may have resulted in over-estimation of poor adherence to treatment. Moreover, the researchers’ assumption that opening the medication monitor box is synonymous with taking the medication may have introduced some inaccuracies into these findings. Despite these limitations and the underuse of more intensive case management in patients with poor adherence, these findings suggest that using medication monitors to deliver electronic drug reminders to patients might improve medication adherence among patients with tuberculosis in China and in other settings.

**Additional Information:**

This list of resources contains links that can be accessed when viewing the PDF on a device or via the online version of the article at http://dx.doi.org/10.1371/journal.pmed.1001876.

- This study is further discussed in a *PLOS Medicine* Perspective by John Metcalfe, Max R. O’Donnell, and David R. Bangsberg
- The World Health Organization provides information (in several languages) on tuberculosis and on its Directly Observed Treatment Short Course (DOTS) strategy; the *Global Tuberculosis Report 2014* provides information about tuberculosis around the world
- The Stop TB Partnership is working towards tuberculosis elimination and provides personal stories about tuberculosis (in English and Spanish); the Tuberculosis Vaccine Initiative (a not-for-profit organization) also provides personal stories about tuberculosis
- The US Centers for Disease Control and Prevention provides information about tuberculosis and about treatment for tuberculosis (in English and Spanish)
- The US National Institute of Allergy and Infectious Diseases also has detailed information on all aspects of tuberculosis
- MedlinePlus has links to further information about tuberculosis (in English and Spanish)
- More information about this trial is available

## Introduction

In 2013, China ranked second in the world in number of tuberculosis (TB) cases, accounting for 11% of the estimated 9 million global cases [1]. Implementation of the Directly Observed Treatment, Short Course (DOTS) strategy started in 1992 and covered the entire country by 2005 [2]. Initially, the use of directly observed treatment (DOT) by health care workers was the primary approach to ensure TB treatment adherence. Over time, because of difficulties in carrying out DOT in many parts of the country, national TB control policies also permitted self-administered treatment and treatment monitored by family members. Over half of TB patients now receive self-administered treatment [3]. In the 2010 National Tuberculosis Prevalence Survey, 20% of TB patients treated by the public health system—using national TB case-management approaches—were lost to follow-up or were not taking their medications regularly [4]. Thus, more effective case-management approaches are needed in China.

Electronic reminders and monitoring have been used in several disease conditions to improve medication adherence. The potential of mobile phone technology to improve the quality and delivery of health care, including diagnosis, treatment adherence, and data collection, has been recognised [5,6]. Mobile phone text messaging has been shown to improve adherence to antiretroviral treatment and outcomes in HIV-positive patients [7]. However, aside from in a small-scale pilot study [8], the use of text messaging has not been rigorously evaluated in TB patients.

Electronic medication packaging (EMP) devices can remind patients to take medications on time, monitor time of drug intake, and alert health care workers to patients who have missed doses [9,10]. The current evidence supporting the use of these devices is limited [10]. In fact, no study to our knowledge has properly evaluated the use of EMP devices in TB patients. Using adherence data from medication monitor boxes (medication monitors) to select less adherent patients for counselling or more intensive forms of case management has been suggested but not studied [11].

To evaluate the use of electronic reminders to improve medication adherence in TB patients, we conducted a cluster-randomised controlled trial to assess the effectiveness of three case-management approaches—using reminders via text messaging, a medication monitor, or both—compared to the standard of care in China.

## Methods

### Ethical Approval

The study was approved by the ethics committees of the Chinese Center for Disease Control and Prevention (201008) and the London School of Hygiene & Tropical Medicine (5704). All patients provided written consent prior to inclusion in the study.

### Study Design

This study was a pragmatic cluster-randomised trial with one control and three intervention arms. New pulmonary TB patients, starting on standard 6-mo short-course chemotherapy and managed as outpatients, were recruited into the study. Those in the control arm were managed according to the standard of care of the National Tuberculosis Control Program. Those in the three intervention arms also received reminders to take their medications from text messages via short message service (SMS), a medication monitor, or both. If adherence problems were subsequently detected, more intensive management was recommended. For logistical simplicity, randomisation was conducted at the cluster level.

### Cluster Selection

Clusters were defined as rural counties or urban districts within the provinces of Heilongjiang, Jiangsu, Hunan, and Chongqing—located in northern, eastern, central, and western China, respectively. Each cluster had at least 300 active pulmonary TB patients registered in 2009 (S3 Text). Nine clusters, with a rural to urban ratio of 2:1, were selected from two cities in each province.

### Patient Recruitment

In each cluster, consecutive pulmonary TB patients newly registered at the public health TB clinic were screened for study eligibility. Inclusion criteria included the following: no communication impairment (mental, visual, auditory, or speech), patient at least 18 y old, and patient or family member able to use mobile phone to read SMS text messages and use the medication monitor after training. Because of the nature of the study, interventions were not masked to study staff and patients.

### Randomisation

The 36 clusters were randomised to the four arms by rural/urban stratum and restricted such that each province had at least two clusters in each arm. From 5,000 randomly generated acceptable allocations, one was chosen at random as the final allocation using Stata version 12.0. See S3 Text for further details.

### All Arms

All patients were treated according to National Tuberculosis Control Program guidelines including the use of isoniazid, rifampin, ethambutol, and pyrazinamide for 2 mo, followed by isoniazid and rifampin for 4 mo; the programme used every other day dosing for the entire treatment course. Patients received their blister-pack medications in a medication monitor box that electronically collected the date and time of each opening. In the control and text messaging arms, the medication monitor box was in silent mode and was not used as a reminder tool for patients. At each monthly visit, patients were dispensed enough medications for a 1-mo period.

### Control Arm

At the start of treatment, the doctor and the patient selected one of three treatment monitoring approaches as per National Tuberculosis Control Program protocol: self-administered treatment, treatment supervised by family members, or treatment supervised by health care workers. The local doctor monitoring treatment at the township or village/community level was given 60 renminbi (RMB; equivalent to US$10) for each patient.

### Intervention Arms

As in the control arm, patients and their doctors in the intervention arms selected one of the three treatment monitoring approaches as per National Tuberculosis Control Program protocol. The interventions had three common components: reminders for timely drug intake, reminders for monthly follow-up visits, and a recommendation for doctors to switch patients from self-administered treatment to a more intensive treatment monitoring approach when patients were found to have adherence problems based on data available to the managing doctor (Table 1).

**Table 1:** Description of the three intervention arms.

Intervention Arm	Component		
	Reminding Patient to Take Medication	Reminding Patient of the Monthly Dispensing Visit^1^	Assessment of Adherence by Doctor at the Monthly Dispensing Visit
**Text messaging only**	There is an agreed time (based on patient preference) for the medication to be taken. Up to three SMS reminders are sent to the patient on the day of medication, depending on whether the patient replies or not. These reminders are sent at the agreed time medication is to be taken and subsequently at 12 noon and 6 p.m. if no reply is received. SMS text is “please take the medication on time” and is the same for each reminder. The patient is expected to reply by SMS with or without text. Once a reply has been received, the reminders stop for that day.	SMS reminder sent 4, 3, 2, and 1 d before the scheduled monthly follow-up visit.	Adherence patterns based on patient interview, pill count from medication monitor box, and SMS feedback. Recommended that: • If 3–6 doses were missed, patient is switched “intensive management”, in which village/community doctors visited the patient once a week for the rest of treatment; • If ≥7 doses were missed or 3–6 doses missed in 2 mo, patient is switched to DOT, with each dose of treatment supervised by the village/community doctor. Incentives (5 RMB/patient visit) were paid to village/community doctors^2^ for intensive management of non-adherent patients.
**Medication monitor only**	There is an agreed time (based on patient preference) for the medication to be taken. If the box is not opened at that time, there are up to eight further reminders (bleep), taking place at 5 min, 20 min, 30 min, 1 h, 2 h, 4 h, 6 h, and 8 h after the agreed time. Once the box has been opened, the reminders stop for that day.	Medication box reminder (human voice) 4, 3, 2, and 1 d before the scheduled monthly follow-up visit.	Adherence patterns based on patient interview, pill count from medication monitor box, and electronic data on dates and times of opening of the medication monitor box. Intensive management/DOT initiation and incentives as above.
**Combined (text messaging and medication monitor)**	A combination of the SMS and medication monitor reminders, as described above.	A combination of the SMS and medication monitor reminders, as described above.	Adherence patterns based on patient interview, pill count from medication monitor box, and electronic data on dates and times of opening of the medication monitor box. Intensive management/DOT initiation and incentives as above.

^1^In all three intervention arms and in the control arm there is a National Tuberculosis Control Program requirement for the managing doctor to contact the patient after 3 d following a missed visit, using all available contact methods.

^2^No incentives were paid to township doctors (urban).

In the text messaging and combined arms, a text message reminded patients to take their medication at the time previously agreed on with the patient. If patient did not reply to the text message, another two text messages would be sent later in the day. Once the patient replied to the SMS reminder, with or without text, the reminders were stopped for that day. Similarly, in the medication monitor and combined arms, an audio reminder from the medication monitor box reminded patients to take their medication. If the patient did not open the medication monitor by a pre-specified time, up to eight additional reminders sounded. Once the box was opened, the reminders were stopped for that day. In all three intervention arms, patients received four reminders to attend the monthly dispensing visit (Table 1).

At each monthly follow-up visit, the managing doctor evaluated adherence patterns. Missed doses were defined as the larger of (1) missed doses based on pill count or (2) missed doses from missing SMS reply (in the text messaging only arm) or from failure to open the medication monitor box (in the other two intervention arms). If the patient reported any equipment malfunction or operation error during the previous month, the number of missed doses was based on pill count only.

If 1–2 doses were missed in the previous month, we recommended the doctor counsel the patient on the importance of adherence to medication but allowed self-administered treatment to continue. If 3–6 doses were missed, we recommended the doctor switch the patient to “intensive management”, in which township or village/community doctors visited the patient twice a month or once a week, respectively, for the rest of treatment. If seven or more doses were missed the previous month or if 3–6 doses were missed in two prior months, we recommended the doctor switch the patient to DOT, with each dose of treatment supervised by the township or village/community doctor. The local doctors monitoring treatment at the township or village/community level were given 5 RMB (US$0.8) every time they made a visit to a patient as part of the intensive management or DOT, in addition to the 60 RMB (US$10) they received for every patient.

### Study Endpoints

All study endpoints were measured at the individual level. The primary study endpoint for treatment adherence was the percentage of patient-months where at least 20% of doses (equivalent to missing three of 15 doses) were missed (“poor adherence”). The secondary treatment adherence endpoints were (1) percentage of patient-months where at least 47% of doses (equivalent to seven of 15 doses) were missed, (2) percentage of total doses missed over the 6 mo of treatment, (3) binary categorisation of secondary endpoint 2 as <10% versus ≥10% of total doses missed (National Tuberculosis Control Program definition of non-adherent), and (4) percentage of patient-months on TB treatment where at least 20% of doses were missed based on pill count only.

Measurement of the adherence endpoints utilised the same data for all four study arms and included data from the medication monitor box, downloaded into a database when patients returned for their monthly medication refill. All adherence endpoints, except secondary endpoint 4, measured the number of missed doses per month as the larger of the number of missed doses from pill count or the number of failures to open the medication monitor box. A month was defined as the number of days between successive appointments, typically 30 d, during which 15 doses should have been taken, but this was adjusted for early/late or missed visits and reduced by the number of days a patient was hospitalised or temporarily discontinued treatment on doctor’s recommendation. Data were censored when a patient died, moved, or permanently discontinued treatment based on a doctor’s decision. For those who were lost to follow-up during treatment, we assumed no drug intake (100% non-adherence) for the period from the date of being lost to follow-up to the date when they should have completed treatment. We also conducted a post hoc sensitivity analysis censoring adherence measurement at the time of loss to follow-up.

The secondary TB treatment outcome endpoints, following standard WHO definitions, were (1) poor treatment outcome, defined as death, treatment failure, or patient loss to follow-up and (2) patient loss to follow-up (S3 Text). Routinely recorded data reported to the National Tuberculosis Control Program were used to define TB treatment outcomes. We also included as a secondary endpoint 2-mo smear conversion among those smear-positive at enrolment.

### Sample Size

Sample size calculations were based on a binary endpoint of non-adherence and took into account the study design [12]. Assuming nine clusters per arm, a two-sided type I error of 5%, and a percentage with non-adherence in the control arm of 30%, 110 TB patients per cluster would be required to detect a 40% reduction in the endpoint in the intervention arm with power of 90% and coefficient of variation of 0.25. The sample size was adjusted to 116 per cluster assuming 5% missing endpoint data. An additional power calculation is summarised in S3 Text.

### Analysis

Analysis of all endpoints used standard methods for a small number of clusters [12], accounting for the stratified design and giving each cluster equal weight (S3 Text). Patients who were reassessed as not having TB by the managing doctor or who were switched to a different treatment model within the first month (due to hospitalisation or travel) were excluded from all analyses. Pre-specified sub-group analyses for the primary endpoint were by age group, literacy, gender, and rural/urban setting.

There were problems with loose batteries in some of the medication monitors, resulting in a power outage during which data on box openings were not captured. The problem could be easily fixed by the patient or the doctor when they became aware of the problem We conducted a post hoc stratified analysis separating patient-months into those that had a record of a medication monitor problem and those that did not (S3 Text).

Analysis was conducted using Stata version 13.

## Results

### Study Population

**Fig 1:**
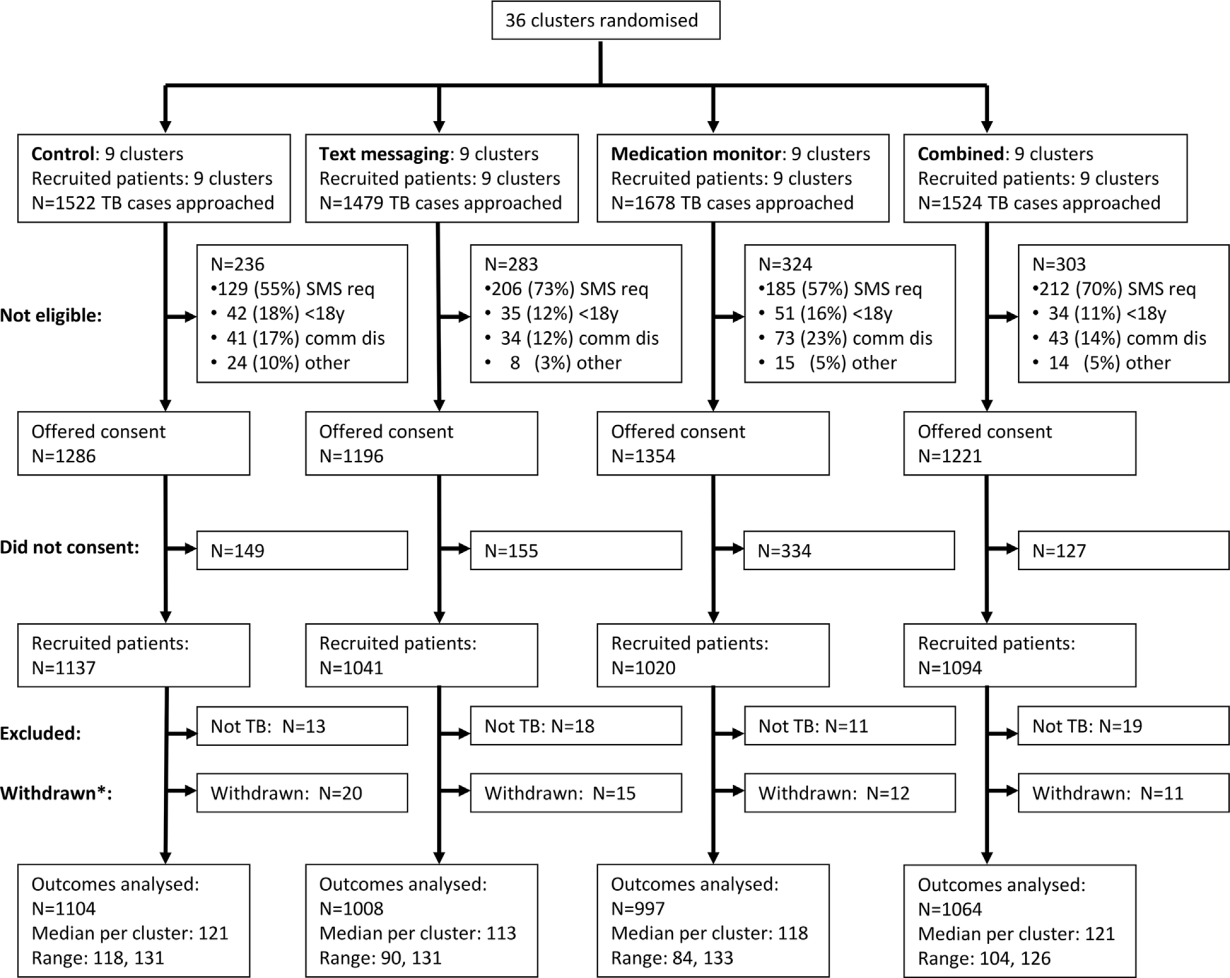
Cluster-level CONSORT diagram. Reasons for non-eligibility: SMS req = unable to use mobile phone after training; <18y = less than 18 y of age; comm dis = communication disability. *Withdrew from the study but continued treatment in the local Center for Disease Control and Prevention.

From 1 June 2011 to 7 March 2012, 6,203 pulmonary TB patients were screened in the 36 clusters, and 5,057 (81.5%) met enrolment criteria, of whom 4,292 (84.9%) gave informed consent. Of these, 61 (1.4%) were reassessed as not having TB by their managing doctor, and 58 (1.4%) were withdrawn from the study as they had switched to a different treatment model within the first month (due to hospitalisation or travel) and were therefore excluded from all analyses (Fig 1; S1 Table). Therefore, 4,173 patients were included in the analysis (Fig 1). There was some variation between arms in the percentages analysed among those screened: 72.5%, 68.2%, 59.4%, and 69.8% in the control, text messaging, medication monitor, and combined arms, respectively (Fig 1).

Overall, 71.0% of participants were male, median age was 43 y (inter-quartile range [IQR] 29 to 56 y), 56.0% were farmers, 7.9% were illiterate, median household income was 20,000 RMB (IQR 10,000 to 30,000 RMB), and 36.3% were smear positive (Table 2). There was some baseline imbalance by study arm for occupation, education level, income, and local residency.

**Table 2:** Characteristics at start of tuberculosis treatment for patients enrolled in the four study arms of the study (*n* = 4,173).

Characteristic	Subcategory	Control Arm (*n* = 1,104)		Text Messaging Arm (*n* = 1,008)		Medication Monitor Arm (*n* = 997)		Combined Arm (*n* = 1,064)	
		Percent	*n*	Percent	*n*	Percent	*n*	Percent	*n*
**Male**		70.1%	774	71.3%	719	71.1%	709	71.6%	762
**Age (years)**	<30	30.2%	333	23.3%	235	23.1%	230	24.2%	258
	30–39	16.0%	177	19.0%	192	11.5%	115	16.8%	179
	40–59	39.1%	432	41.1%	414	39.1%	390	41.2%	438
	60+	14.7%	162	16.6%	167	26.3%	262	17.8%	189
**Farmer**		48.9%	540	60.7%	612	66.0%	658	49.5%	527
**Education level**	Illiterate	7.3%	81	5.3%	53	11.2%	112	8.0%	85
	Lower middle	62.8%	693	75.8%	764	66.7%	665	63.3%	674
	Upper middle	17.6%	194	12.9%	130	13.2%	132	19.1%	203
	University	12.3%	136	6.1%	61	8.8%	88	9.6%	102
**Marital status**	Not married	23.9%	264	15.8%	159	18.3%	182	19.0%	202
	First marriage	69.7%	770	77.8%	784	76.0%	758	73.0%	777
	Other	6.3%	70	6.4%	65	5.7%	57	8.0%	85
**Local resident**		84.3%	931	92.4%	931	97.8%	975	91.5%	974
**Income ≥ 20,000 RMB** ^1^		41.1%	454	27.9%	281	28.2%	281	26.0%	277
**Distance to TB clinic (km)**	<10	23.3%	257	24.9%	251	17.5%	174	35.9%	382
	10–29	38.6%	426	42.0%	423	37.0%	369	32.1%	342
	20–39	18.1%	200	12.1%	122	15.6%	156	13.3%	141
	≥40	20.0%	221	21.0%	212	29.9%	298	18.7%	199
**Distance to local village/township doctor (km)**	≤1	66.6%	735	49.5%	499	63.5%	633	66.1%	703
	2	21.8%	241	32.0%	323	21.1%	210	20.1%	214
	>2	11.6%	128	18.5%	186	15.4%	154	13.8%	147
**Smear positive**		33.8%	373	38.0%	383	39.0%	389	34.6%	368

Table excludes 61 patients who were reassessed as not having TB by their managing doctor and 58 patients who were withdrawn from the study as they switched to a different treatment model within the first month (due to hospitalisation or travel).

^1^Over last calendar year.

### Endpoints

#### Primary study endpoint

**Fig 2:**
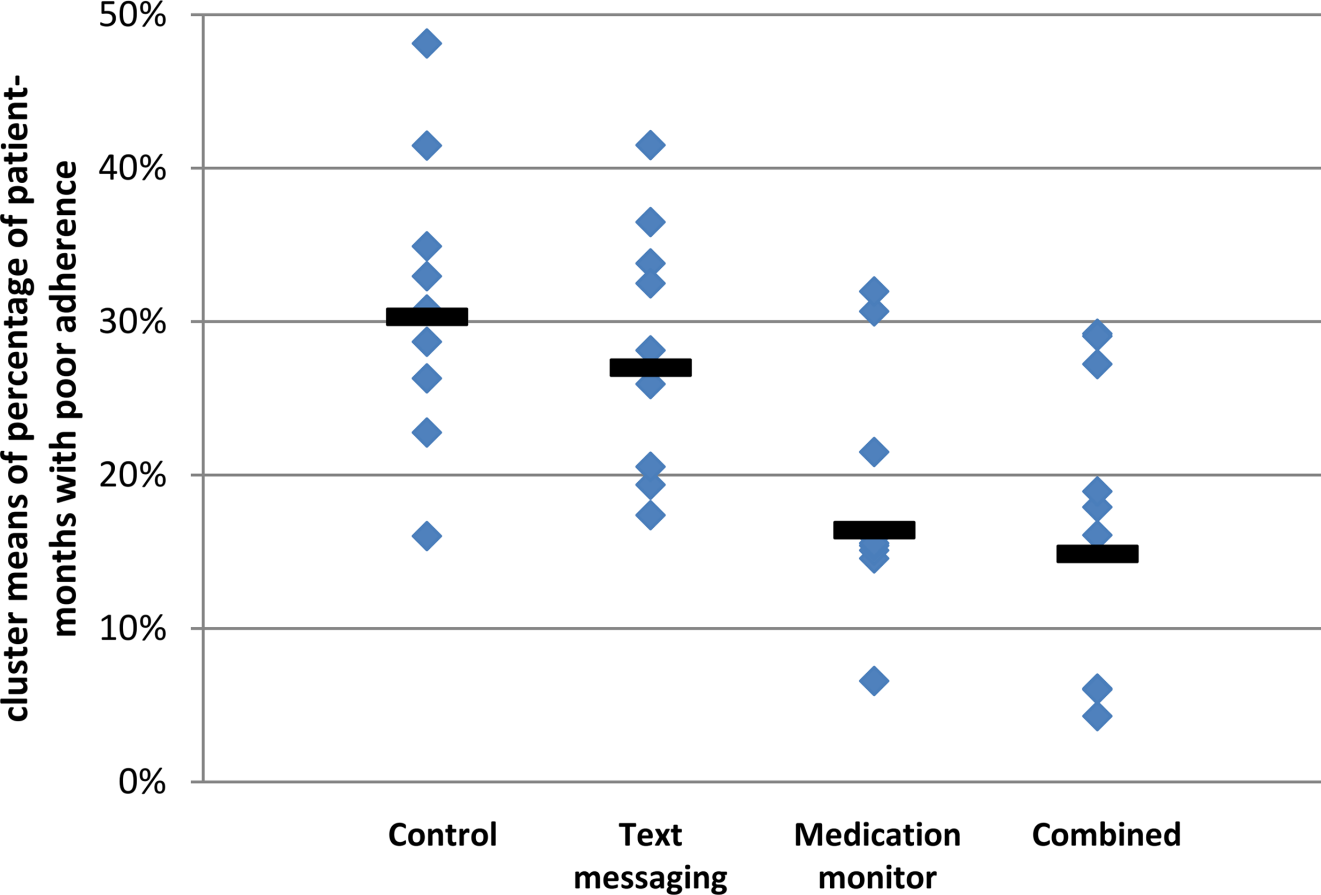
Primary endpoint of poor tuberculosis treatment adherence by study arm. Solid bars represent geometric means of cluster-level proportions.

The cluster geometric mean of the percentage of patient-months on TB treatment where at least 20% of doses were missed was 29.9% in the control arm (range 16.0%–48.1%; Fig 2; S2 Table); in comparison, this percentage was 27.3% in the text messaging arm (adjusted mean ratio [aMR] 0.94, 95% CI 0.71, 1.24; Table 3), 17.0% in the medication monitor arm (aMR 0.58, 95% CI 0.42, 0.79), and 13.9% in the combined arm (aMR 0.49, 95% CI 0.27, 0.88). There were no differences in the mean ratios (MRs) for the primary endpoint when stratifying by age, literacy, or gender (S3 Table). There was an indication that the reduction in poor adherence seen in the medication monitor arm compared to the control arm was only in the rural stratum (MR 0.43 for rural and 1.06 for urban, *p*-value for effect modification 0.011). The coefficient of variation for the primary endpoint among control clusters was 0.24.

**Table 3:** Effectiveness of interventions on tuberculosis treatment adherence and treatment outcomes endpoints.

Endpoint and Study Arm	Number of Patients	Geometric Mean of Cluster-Level Endpoint	Unadjusted Analysis		Adjusted Analysis^1^	
			MR (95% CI)	*p*-Value	MR (95% CI)	*p*-Value
**Primary endpoint—percentage of patient-months with at least 3/15 doses missed** ^2^ ^,^ ^3^ ^,^ ^4^						
Control	1,091	29.9%	1		1	
Text messaging	996	27.3%	0.91 (0.66, 1.25)	0.536	0.94 (0.71, 1.24)	0.622
Medication monitor	992	17.0%	0.57 (0.40, 0.81)	0.004	0.58 (0.42, 0.79)	0.002
Combined	1,059	13.9%	0.46 (0.25, 0.86)	0.018	0.49 (0.27, 0.88)	0.020
**Percentage of months with at least 7/15 doses missed** ^2^ ^,^ ^3^						
Control	1,091	18.9%	1		1	
Text messaging	996	17.8%	0.94 (0.63, 1.41)	0.744	0.96 (0.67, 1.38)	0.808
Medication monitor	992	11.1%	0.59 (0.38, 0.91)	0.022	0.60 (0.40, 0.89)	0.015
Combined	1,059	9.4%	0.50 (0.26, 0.94)	0.034	0.52 (0.28, 0.97)	0.042
**Percentage of total doses missed** ^2^ ^,^ ^3^						
Control	1,091	22.6%	1		1	
Text messaging	996	20.7%	0.92 (0.66, 1.28)	0.584	0.94 (0.70, 1.26)	0.649
Medication monitor	992	13.9%	0.61 (0.44, 0.86)	0.008	0.62 (0.46, 0.84)	0.004
Combined	1,059	11.4%	0.51 (0.28, 0.92)	0.029	0.53 (0.29, 0.95)	0.034
**At least 10% of total doses missed** ^2^ ^,^ ^3^						
Control	1,091	57.4%	1		1	
Text messaging	996	54.7%	0.95 (0.74, 1.23)	0.690	0.97 (0.77, 1.23)	0.807
Medication monitor	992	38.7%	0.67 (0.50, 0.90)	0.011	0.68 (0.52, 0.89)	0.008
Combined	1,059	31.0%	0.54 (0.30, 0.96)	0.037	0.56 (0.33, 0.97)	0.041
**Percentage of patient-months with at least 3/15 doses missed (using pill count only)** ^3^						
Control	1,091	9.2%	1		1	
Text messaging	996	3.8%	0.41 (0.20, 0.87)	0.023	0.39 (0.18, 0.83)	0.018
Medication monitor	992	5.5%	0.60 (0.33, 1.08)	0.084	0.58 (0.35, 0.96)	0.037
Combined	1,059	6.4%	0.70 (0.34, 1.45)	0.307	0.67 (0.31, 1.47)	0.294
**Poor treatment outcome (treatment failure, death, or patient loss to follow-up)** ^5^						
Control	1,066	8.6%	1		1	
Text messaging	966	3.9%	0.45 (0.18, 1.16)	0.092	0.44 (0.17, 1.13)	0.084
Medication monitor	955	6.1%	0.70 (0.32, 1.53)	0.264	0.71 (0.33, 1.51)	0.346
Combined	992	8.8%	1.01 (0.46, 2.22)	0.973	1.00 (0.45, 2.20)	0.991
**Patient loss to follow-up** ^6^						
Control	1,057	8.5%	1		1	
Text messaging	954	3.6%	0.42 (0.18, 1.00)	0.050	0.42 (0.18, 0.98)	0.046
Medication monitor	946	5.0%	0.58 (0.23, 1.51)	0.243	0.61 (0.25, 1.51)	0.264
Combined	982	7.6%	0.90 (0.38, 2.08)	0.783	0.90 (0.38, 2.09)	0.784

^1^Adjusted for individual-level variables of gender, age group, occupation (farmer or not), local resident or not, distance to nearest TB clinic, education level, income category, and smear result at start of treatment, and for the cluster-level variable of pre-randomisation stratum (rural/urban).

^2^Doses missed based on the larger of missed doses from (1) pill count or (2) the number of failures to open the medication monitor.

^3^Excludes 35 patients who had no adherence data (by arm: 13 in control, 12 in text messaging, five in medication monitor, and five in combined).

^4^Data collected monthly, then aggregated at the patient level as a proportion. The arithmetic means of these proportions were used to produce a cluster-level summary. Finally, the geometric mean (as a log transformation of the cluster-level summaries; see S1 Text) of the nine cluster-level summaries was used in each arm as the summary in this table. The month-level data by arm, ignoring clustering at the patient and cluster levels, are as follows: control arm—1,834/6,013 poor adherence patient-months (30.5%); text messaging arm—1,518/5,284 poor adherence patient-months (28.7%); medication monitor arm—943/5,430 poor adherence patient-months (17.4%); combined arm—981/5,782 poor adherence patient-months (17.0%).

^5^Excludes 188 patients with outcome of side effect on treatment, resulting in an extension on TB treatment and the final outcome not being documented (by arm: 38 in control, 42 in text messaging, 41 in medication monitor, and 67 in combined), five patients who transferred to another clinic (all in combined arm; unknown outcome in new clinic), and one patient with missing outcome (in medication monitor arm). The numbers of patients with a poor treatment outcome by arm, ignoring cluster, are as follows: control arm—121/1,066; text messaging arm—53/966; medication monitor arm—68/955; combined arm—99/992.

^6^Excludes 188 patients with outcome of side effect on treatment, resulting in an extension on TB treatment and the final outcome not being documented (by arm: 38 in control, 42 in text messaging, 41 in medication monitor, 67 in combined), 13 patients with treatment failure (by arm: three in control, six in text messaging, one in medication monitor, three in combined), 27 deaths (by arm: six in control, six in text messaging, eight in medication monitor, seven in combined), five patients who transferred to another clinic (all in combined arm; unknown outcome in new clinic), and one patient with missing outcome (medication monitor arm). The numbers of patients lost to follow-up by arm, ignoring cluster, are as follows: control arm—112/1,057; text messaging arm—41/954; medication monitor arm—59/946; combined arm—89/982.

#### Secondary study endpoints

There were similar reductions in the intervention arms versus the control arm in the percentage of months with at least 47% of doses missed (equivalent to 7/15 doses), the percentage of doses missed over the whole treatment period, and the percentage of patients who missed at least 10% of their doses, in both unadjusted and adjusted analyses (Table 3). The percentage of person-months with at least 20% of doses missed as judged by pill count only was much lower than that judged by both pill count and medication monitor data. This secondary endpoint was reduced by 33%–61% in the three intervention arms compared to the control arm, but this reduction was mostly driven by the imputation of months with 100% non-adherence following loss to follow-up (Table 3).

The text messaging arm had a lower patient loss to follow-up and occurrence of poor treatment outcome than the control arm. Modest reductions in patient loss to follow-up were also seen for the medication monitor and combined arms, though confidence intervals for the effect estimates included one. A post hoc sensitivity analysis that censored adherence measurement at the time of loss to follow-up showed a strengthening of the evidence for a reduction in poor adherence as measured by pill count in the three intervention arms, but otherwise very similar results (S4 Table). There were too few patients with data on sputum conversion at 2 mo for a formal analysis (summary data in S3 Text).

Problems with the medication monitor box, recorded either by the doctor at the monthly visit or by the medication monitor as power interruption, were more common in the medication monitor (49.4% of patients; Table 4) and combined arms (48.0%) than in the control (17.8%) or text messaging arms (16.7%). Stratified analysis of the primary endpoint by noted medication monitor problems showed that the reduction in poor adherence persisted in the medication monitor and combined arms regardless of whether there were power problems (S5 Table).

**Table 4:** Intervention process data and medication monitoring data by study arm.

Process Measure	Control Arm (*n* = 1,104)		Text Messaging Arm (*n* = 1,008)		Medication Monitor Arm (*n* = 997)		Combined Arm (*n* = 1,064)	
	Percent^1^	*n*	Percent^1^	*n*	Percent^1^	*n*	Percent^1^	*n*
**Patients with a medication monitor problems**								
Reported by doctor	7.4%	82	9.0%	91	44.2%	441	41.1%	437
Recorded by medication monitor^2^	12.0%	132	9.8%	99	20.9%	208	21.4%	228
Any problem^3^	17.8%	196	16.7%	168	49.4%	492	48.0%	511
**Patients with mobile phone problems**								
Reported by doctor			56.5%	569			27.3%	290
**Intensive management**								
Should start^4^			4.1%	41	4.3%	43	4.4%	47
Started			4.0%	40	3.2%	32	4.1%	44
**DOT**								
Should start^4^			0.8%	8	1.3%	13	1.4%	15
Started			0.8%	8	0.9%	9	0.9%	10

^1^Percentage denominator is total number of patients in arm.

^2^An incorrect date was recorded by the medication monitor, indicating the power had failed and then been resolved without resetting the internal clock to the correct date.

^3^Reported by doctor or recorded by medication monitor.

^4^According to information available to the patient’s dispensing doctor.

### Process Measures

Similar percentages of patients in the three intervention arms were switched to intensive management (3.2%–4.1%) and DOT (0.8%–0.9%) (Table 4). Based on combining the data from pill counts and the medication monitor, the percentages of patients who should have been switched to intensive management or DOT, were 26.3% and 35.4%, respectively, in the text messaging arm, 16.2% and 24.1% in the medication monitor arm and 16.0% and 20.1% in the combined arm.

Minor problems with the mobile phones used to receive text messages were also common and were reported by 56.5% of those in the text messaging arm and 27.3% of those in the combined arm (Table 4). These problems included incorrect usage of the phone by the patient (42.0%), network failure (21.1%), and no money on the phone account (14.9%). Problems with the medication monitor or phone were resolved in 88.7% of occurrences (S6 Table).

## Discussion

Our study found that the use of a medication monitor to remind TB patients to take their drugs reduced poor medication adherence by 40%–50% compared to the standard of care in China’s National Tuberculosis Control Program. This reduction was seen for all TB treatment adherence measures in this study. The use of text messaging did not reduce poor medication adherence but did reduce patient loss to follow-up by 58%. The use of a medication monitor alone resulted in a smaller, and not statistically significant, reduction in patient loss to follow-up compared to control; however, the study was not powered for this treatment outcome.

Even though many types of EMP devices exist and have been used for different disease conditions, a recent systematic review concluded that there were limited data supporting their use in improving adherence [9]. This study is the first to our knowledge to utilise a randomised trial design to demonstrate the effectiveness of an EMP device in improving medication adherence in TB patients. The use of our medication monitor box was integrated into the public health management of TB treatment. Such integration seems to be more frequently associated with improved medication adherence [9,10]. As the largest study to date, to our knowledge, to evaluate an EMP device for any disease condition, this study provides important evidence supporting the use of EMP to improve medication adherence.

Our results demonstrate that text messaging did not reduce poor medication adherence among TB patients. This contrasts with available evidence supporting the use of text messaging among HIV patients on antiretroviral therapy [7], which led to a strong recommendation from WHO for its use [13]. However, not all text messages are effective. There was a trend for greater effects of an intervention with texts that were less frequent than daily and with more personalised messages [7]. Frequent medication reminders using text messages can result in user fatigue. And some experts suggest that the most important factor is whether patients feel cared for, not the length or frequency of the text messages. Perhaps the lack of a more personalised engagement, the didactic nature of our messages, multiple messages per day, and the SMS message being received when the patient was not in close proximity to his/her medication all contributed to the failure to reduce poor adherence. There has been recent interest in using mobile phone technology to improve adherence to TB medication [5,14] and TB treatment outcomes [15], though, as yet, few studies have reported their findings [16].

In our intervention arms, we recommended that doctors switch patients to intensive patient management or DOT when adherence problems were documented. However, this rarely happened, despite data suggesting that a substantial percentage of patients should have switched. The trial was designed to be pragmatic, and so we did not enforce the initiation of more intensive management or DOT. Because problems with medication monitors, mobile phones, or their use were frequently reported, it is possible that doctors largely chose to ignore the electronic adherence data when deciding whether to switch patients to more intensive case-management approaches. In addition, doctors may not have had sufficient financial incentives to carry out more intensive case management.

Even though more intensive case-management approaches were underutilised in the presence of recorded treatment non-adherence, we still observed better adherence in the medication monitor and combined arms. This suggests that the use of a medication monitor to remind patients to take their medications can improve treatment adherence by itself. If information on poor treatment adherence had been used by providers to switch patients to more intensive case-management approaches, as intended, it is likely we would have seen an even greater reduction in poor treatment adherence with the use of medication monitors.

Interestingly, text messaging reduced the risk of patient loss to follow-up. Perhaps text messaging is an effective approach to remind patients of follow-up visits and resulted in better attendance at monthly visits. However, a recent meta-analysis suggests that the effectiveness of SMS reminders for appointments is modest at best and not more effective than other types of reminders [6,17].

The differences in the effects of the interventions in terms of adherence and treatment outcome endpoints suggest these do not correlate well. However, adherence is complex, and a recent taxonomy divides it into three constructs—initiation (patient takes the first dose), implementation (measure of how patient’s actual dosing history corresponds to the prescribed dosing regimen from initiation until the last dose is taken), and discontinuation (patient stops taking the prescribed medication) [18]. Given that our primary adherence endpoint is predominantly “implementation”, the seemingly discrepant results are not surprising; the evidence did not change when the primary endpoint was restricted to “implementation” only, in a post hoc sensitivity analysis. We defined poor adherence based on a threshold of 20% missed doses, a threshold commonly used in other disease areas [19].

Our study had several limitations. First, the battery problems with the medication monitors in our study led to loss of data in some patients, potentially resulting in an over-estimation of poor adherence. However, when we performed a stratified analysis using patient-months with or without this problem, we found similar reductions in poor treatment adherence. Second, more intensive case-management approaches were underutilised, possibly because doctors disregarded information from the medication monitor or SMS feedback. In addition, the financial incentives given to the doctors to perform more intensive management may have been inadequate. Third, for the adherence endpoints, we assumed that opening the medication monitor box was synonymous with drug intake, which may not have been the case, though our measure of poor adherence using a combination of this and pill count is arguably more robust than pill count alone. Pill counts have often been shown to under-report poor adherence or non-adherence [18], as is also shown in our study, where the geometric mean for the adherence endpoint measured using pill count only was lower than that of the primary endpoint. Further, data from a separate study indicated high correlation between adherence measured by medication monitor and rifampicin detected in urine [20]. Other limitations included differences in percentages enrolled by study arm, baseline imbalance of some factors, the unmasked nature of the trial, and the study not being powered for the treatment outcome endpoints. In spite of these limitations, this is the largest study to date, to our knowledge, to evaluate the use of text messaging or medication monitors to improve medication adherence in TB patients. As a pragmatic trial, implemented by the National Tuberculosis Control Program and mimicking real-world conditions, this study has produced useful lessons for future study designs.

The use of a medication monitor as a reminder for drug intake in combination with the identification of patients requiring more intensive management has been suggested as an approach for improving TB treatment adherence [11]. This is the first study to our knowledge to rigorously evaluate such an approach. Based on our results, the use of a medication monitor shows great promise. In a setting such as China, where universal use of DOT is not feasible, innovative approaches that help patients adhere to TB treatment are needed. The development of a low-cost and reliable medication monitor, as well as evidence that its use can improve clinical outcomes, could enable widespread use of medication monitors in national TB control programmes.

## Supporting Information

S1 Table Characteristics at start of tuberculosis treatment for patients in the four study arms who were withdrawn from the study due to hospitalisation or travel (*n* = 58). (DOCX)

S2 Table Primary outcome of poor adherence (defined as percentage of patient-months in which a patient missed at least 20% of doses) by cluster, study arm, and rural/urban stratum. (DOCX)

S3 Table Pre-specified sub-group analyses of the primary endpoint (percentage of patient-months with at least 20% doses missed). (DOCX)

S4 Table Effectiveness of interventions for endpoints of tuberculosis treatment adherence based on adherence measures before imputation of non-adherence for those who were lost to follow-up (post hoc sensitivity analysis). (DOCX)

S5 Table Sensitivity analysis of the primary endpoint (percentage of patient-months with at least 20% doses missed): post hoc sub-group analysis. (DOCX)

S6 Table Problems with medication monitors and mobile phones by study arm. (DOCX)

S1 Text Trial protocol. (DOCX)

S2 Text CONSORT checklist. (DOCX)

S3 Text Trial methods and results. (DOCX)

## Abbreviations

aMR: adjusted mean ratio
DOT: directly observed treatment
EMP: electronic medication packaging
IQR: inter-quartile range
MR: mean ratio
RMB: renminbi
SMS: short message service
TB: tuberculosis
