# Supplementary material for: Effectiveness of Electronic Reminders to Improve Medication Adherence in Tuberculosis Patients: A Cluster-Randomised Trial
Source: PLoS Med. 2015 Sep 15;12(9):e1001876. doi: 10.1371/journal.pmed.1001876 (PMC4570796; doi:10.1371/journal.pmed.1001876)
Supplement: S1 Table — (DOCX) [file pmed.1001876.s001.docx]

**S1 Table. Characteristics at start of tuberculosis treatment by the four study arms for patients withdrawn from the study due to hospitalisation or travel (n=58)**

|  |  | Control | | Text messaging | | Medication Monitor | | Combined | |
| --- | --- | --- | --- | --- | --- | --- | --- | --- | --- |
| Consented |  | n=20 |  | n=15 |  | n=12 |  | n=11 |  |
|  |  | % | n | % | n | % | n | % | n |
| Male |  | 55.0% | 11 | 66.7% | 10 | 66.7% | 8 | 63.6% | 7 |
|  |  |  |  |  |  |  |  |  |  |
| Age (years) | <30 | 25.0% | 5 | 40.0% | 6 | 25.0% | 3 | 0.0% | 0 |
|  | 30-39 | 30.0% | 6 | 6.7% | 1 | 16.7% | 2 | 27.3% | 3 |
|  | 40-59 | 40.0% | 8 | 33.3% | 5 | 25.0% | 3 | 45.5% | 5 |
|  | 60+ | 5.0% | 1 | 20.0% | 3 | 33.3% | 4 | 27.3% | 3 |
|  |  |  |  |  |  |  |  |  |  |
| Farmer |  | 30.0% | 6 | 46.7% | 7 | 58.3% | 7 | 81.8% | 9 |
|  |  |  |  |  |  |  |  |  |  |
| Education status | Illiterate | 0.0% | 0 | 6.7% | 1 | 16.7% | 2 | 9.1% | 1 |
|  | Lower middle | 80.0% | 16 | 60.0% | 9 | 66.7% | 8 | 90.9% | 10 |
|  | Upper middle | 10.0% | 2 | 26.7% | 4 | 0.0% | 0 | 0.0% | 0 |
|  | University | 10.0% | 2 | 6.7% | 1 | 16.7% | 2 | 0.0% | 0 |
|  |  |  |  |  |  |  |  |  |  |
| Marital status | Not married | 15.0% | 3 | 20.0% | 3 | 25.0% | 3 | 18.2% | 2 |
|  | First marriage | 75.0% | 15 | 73.3% | 11 | 66.7% | 8 | 72.7% | 8 |
|  | Other | 10.0% | 2 | 6.7% | 1 | 8.3% | 1 | 9.1% | 1 |
|  |  |  |  |  |  |  |  |  |  |
| Local residency |  | 80.0% | 16 | 53.3% | 8 | 100.0% | 12 | 90.9% | 10 |
|  |  |  |  |  |  |  |  |  |  |
| Income ≥20,000 RMB |  | 65.0% | 13 | 46.7% | 7 | 91.7% | 11 | 63.6% | 7 |
|  |  |  |  |  |  |  |  |  |  |
| Distance to TB clinic (km) | <10 | 30.0% | 6 | 13.3% | 2 | 33.3% | 4 | 9.1% | 1 |
|  | 10-29 | 35.0% | 7 | 66.7% | 10 | 25.0% | 3 | 27.3% | 3 |
|  | 20-39 | 10.0% | 2 | 0.0% | 0 | 16.7% | 2 | 27.3% | 3 |
|  | ≥40 | 25.0% | 5 | 20.0% | 3 | 25.0% | 3 | 36.4% | 4 |
|  |  |  |  |  |  |  |  |  |  |
| Distance to Supervision (km) | ≤1 | 60.0% | 12 | 46.7% | 7 | 58.3% | 7 | 36.4% | 4 |
|  | 2 | 35.0% | 7 | 20.0% | 3 | 16.7% | 2 | 27.3% | 3 |
|  | >2 | 5.0% | 1 | 33.3% | 5 | 25.0% | 3 | 36.4% | 4 |
|  |  |  |  |  |  |  |  |  |  |
| Smear positive |  | 45.0% | 9 | 40.0% | 6 | 66.7% | 8 | 63.6% | 7 |
