# Supplementary material for: Effectiveness of Electronic Reminders to Improve Medication Adherence in Tuberculosis Patients: A Cluster-Randomised Trial
Source: PLoS Med. 2015 Sep 15;12(9):e1001876. doi: 10.1371/journal.pmed.1001876 (PMC4570796; doi:10.1371/journal.pmed.1001876)
Supplement: S2 Table — (DOCX) [file pmed.1001876.s002.docx]

**S2 Table. Primary outcome of poor adherence (defined as percentage of months in which a patient missed at least 20% of doses) by cluster, study arm and rural/urban strata.**

| **Strata** | **Cluster number** | **Control** | | **Text messaging** | | **Medication monitor** | | **Combined** | |
| --- | --- | --- | --- | --- | --- | --- | --- | --- | --- |
|  |  | **Number patients** | **Cluster mean #** | **Number patients** | **Cluster mean #** | **Number patients** | **Cluster mean #** | **Number patients** | **Cluster mean #** |
| rural | 1 | 121 | 22.7% | 97 | 17.4% | 109 | 6.6% | 121 | 4.3% |
| rural | 2 | 117 | 26.3% | 110 | 19.4% | 133 | 14.5% | 121 | 6.1% |
| rural | 3 | 119 | 33.0% | 129 | 28.1% | 118 | 15.1% | 116 | 17.9% |
| rural | 4 | 115 | 34.9% | 97 | 33.8% | 116 | 15.4% | 104 | 18.9% |
| rural | 5 | 128 | 41.4% | 122 | 36.5% | 119 | 15.9% | 123 | 27.2% |
| rural | 6 | 124 | 48.1% | 90 | 41.5% | 95 | 21.5% | 113 | 29.2% |
| urban | 7 | 120 | 16.0% | 126 | 20.5% | 119 | 15.5% | 115 | 6.0% |
| urban | 8 | 129 | 28.7% | 130 | 25.9% | 84 | 30.7% | 122 | 16.1% |
| urban | 9 | 118 | 30.8% | 95 | 32.5% | 99 | 32.0% | 124 | 29.0% |
|  |  |  |  |  |  |  |  |  |  |
| Overall |  | 1091 | 31.4% | 996 | 28.1% | 992 | 17.9% | 1059 | 17.2% |
| GM* |  |  | 29.9% |  | 27.3% |  | 17.0% |  | 13.9% |

#Cluster-level arithmetic mean of patient-level proportion of months with poor adherence

* Geometric mean of cluster level means
