# Supplementary material for: Effectiveness of Electronic Reminders to Improve Medication Adherence in Tuberculosis Patients: A Cluster-Randomised Trial
Source: PLoS Med. 2015 Sep 15;12(9):e1001876. doi: 10.1371/journal.pmed.1001876 (PMC4570796; doi:10.1371/journal.pmed.1001876)
Supplement: S3 Table — (DOCX) [file pmed.1001876.s003.docx]

**S3 Table. Pre-specified sub-group analyses of the primary endpoint of the percentage of months with at least 20% doses missed**

|  | **Number of patients** | **Geometric mean of cluster level endpoint** | **Adjusted analysis**^1^ | | **Number of patients** | **Geometric mean of cluster level endpoint** | **Adjusted analysis**^1^ | | **p-value for effect modification** |
| --- | --- | --- | --- | --- | --- | --- | --- | --- | --- |
|  |  |  | **Mean ratio**  **(95% CI)** | **p-value** |  |  | **Mean ratio**  **(95% CI)** | **p-value** |  |
|  | **Age < 40 years** | | | | **Age ≥ 40 years** | | | |  |
| *Control* | 504 | 30.9% | 1 |  | 587 | 27.9% | 1 |  |  |
| *Text messaging* | 423 | 29.9% | 0.98 (0.78, 1.25) | 0.884 | 573 | 24.3% | 0.88 (0.61, 1.28) | 0.490 | 0.347 |
| *Medication monitor* | 344 | 16.4% | 0.54 (0.34, 0.83) | 0.009 | 648 | 16.6% | 0.59 (0.43, 0.82) | 0.004 | 0.409 |
| *Combined* | 435 | 13.6% | 0.46 (0.25, 0.85) | 0.017 | 624 | 13.8% | 0.51 (0.27, 0.94) | 0.033 | 0.384 |
|  | **Illiterate** | | | | **Literate** | | | |  |
| *Control* | 78 | 33.4% | 1 |  | 1013 | 29.6% | 1 |  |  |
| *Text messaging* | 49 | 27.5% | 0.81 (0.35, 1.88) | 0.606 | 947 | 27.4% | 0.95 (0.73, 1.24) | 0.686 | 0.647 |
| *Medication monitor* | 110 | 15.4% | 0.46 (0.27, 0.79) | 0.008 | 882 | 16.6% | 0.58 (0.41, 0.82) | 0.004 | 0.781 |
| *Combined* | 85 | 19.7% | 0.60 (0.31, 1.15) | 0.116 | 974 | 12.7% | 0.45 (0.22, 0.91) | 0.030 | 0.399 |
|  | **Male** | | | | **Female** | | | |  |
| *Control* | 765 | 31.5% | 1 |  | 326 | 25.4% | 1 |  |  |
| *Text messaging* | 709 | 28.6% | 0.93 (0.72, 1.20) | 0.553 | 287 | 23.0% | 0.95 (0.62, 1.46) | 0.809 | 0.856 |
| *Medication monitor* | 704 | 17.7% | 0.57 (0.41, 0.79) | 0.002 | 288 | 14.7% | 0.60 (0.41, 0.89) | 0.014 | 0.713 |
| *Combined* | 757 | 14.4% | 0.48 (0.27, 0.86) | 0.018 | 302 | 12.5% | 0.52 (0.27, 0.99) | 0.048 | 0.508 |
|  | **Rural** | | | | **Urban** | | | |  |
| *Control* | 724 | 34.6% | 1 |  | 367 | 25.2% | 1 |  |  |
| *Text messaging* | 645 | 29.3% | 0.85 (0.58, 1.25) | 0.383 | 351 | 25.8% | 1.12 (0.69, 1.83) | 0.546 | 0.340 |
| *Medication monitor* | 690 | 14.7% | 0.43 (0.29, 0.62) | <0.001 | 302 | 25.1% | 1.06 (0.48, 2.37) | 0.846 | 0.011 |
| *Combined* | 698 | 17.1% | 0.43 (0.20, 0.91) | 0.031 | 361 | 17.3% | 0.63 (0.17, 2.35) | 0.385 | 0.520 |

CI=confidence interval;

^1^ adjusted for individual level variables of gender, age category, occupation, living in household registration place or not, distance from nearest TB clinic and cluster level variable of pre-randomisation strata (rural/urban), excluding sub-group variable.
