# Supplementary material for: Effectiveness of Electronic Reminders to Improve Medication Adherence in Tuberculosis Patients: A Cluster-Randomised Trial
Source: PLoS Med. 2015 Sep 15;12(9):e1001876. doi: 10.1371/journal.pmed.1001876 (PMC4570796; doi:10.1371/journal.pmed.1001876)
Supplement: S4 Table — (DOCX) [file pmed.1001876.s004.docx]

**S4 Table. Effectiveness of interventions for endpoints of tuberculosis treatment adherence based on adherence measures censored at the time of loss to follow-up (post-hoc sensitivity analysis).**

| **Study arm** | **Number of patients** | **Geometric mean of cluster level endpoint** | **Unadjusted analysis** | | | **Adjusted analysis**^1^ | | |
| --- | --- | --- | --- | --- | --- | --- | --- | --- |
|  |  |  | **Mean ratio**  **(95% CI)** | **p-value** | | **Mean ratio**  **(95% CI)** | **p-value** | |
| **Primary endpoint – Percentage of months with at least 3/15 doses missed^2^** | | | | | | | | |
| *Control* | 1076 | 25.4% | 1 | |  | 1 | |  |
| *Text messaging* | 984 | 24.7% | 0.97 (0.71, 1.34) | | 0.865 | 1.00 (0.74, 1.34) | | 0.979 |
| *Medication monitor* | 978 | 13.7% | 0.54 (0.39, 0.75) | | 0.001 | 0.55 (0.41, 0.75) | | 0.001 |
| *Combined* | 1032 | 9.0% | 0.36 (0.18, 0.72) | | 0.007 | 0.38 (0.19, 0.73) | | 0.007 |
| **Percentage of months with at least 7/15 doses missed^2^** | | | | | | | | |
| *Control* | 1076 | 12.9% | 1 | |  | 1 | |  |
| *Text messaging* | 984 | 14.8% | 1.15 (0.75, 1.76) | | 0.487 | 1.16 (0.77, 1.73) | | 0.453 |
| *Medication monitor* | 978 | 7.5% | 0.58 (0.37, 0.92) | | 0.025 | 0.59 (0.38, 0.91) | | 0.021 |
| *Combined* | 1032 | 4.5% | 0.35 (0.16, 0.74) | | 0.010 | 0.38 (0.19, 0.78) | | 0.012 |
| **Percentage of total doses missed^2^** | | | | | | | | |
| *Control* | 1076 | 17.0% | 1 | |  | 1 | |  |
| *Text messaging* | 984 | 17.9% | 1.05 (0.76, 1.46) | | 0.748 | 1.06 (0.78, 1.45) | | 0.671 |
| *Medication monitor* | 978 | 10.3% | 0.60 (0.43, 0.84) | | 0.005 | 0.61 (0.45, 0.82) | | 0.003 |
| *Combined* | 1032 | 6.5% | 0.38 (0.19, 0.78) | | 0.012 | 0.41 (0.21, 0.80) | | 0.013 |
| **At least 10% of total doses missed^2^** | | | | | | | | |
| *Control* | 1076 | 52.0% | 1 | |  | 1 | |  |
| *Text messaging* | 984 | 52.1% | 1.00 (0.77, 1.31) | | 0.989 | 1.02 (0.79, 1.30) | | 0.899 |
| *Medication monitor* | 978 | 34.3% | 0.66 (0.49, 0.89) | | 0.009 | 0.65 (0.49, 0.87) | | 0.006 |
| *Combined* | 1032 | 24.9% | 0.48 (0.25, 0.93) | | 0.033 | 0.50 (0.27, 0.94) | | 0.033 |
| **Percentage of months with at least 3/15 doses missed (using pill count only)** | | | | | | | | |
| *Control* | 1076 | 3.5% | 1 | |  | 1 | |  |
| *Text messaging* | 984 | 1.2% | 0.33 (0.15, 0.76) | | 0.012 | 0.33 (0.14, 0.75) | | 0.012 |
| *Medication monitor* | 978 | 1.6% | 0.46 (0.23, 0.95) | | 0.036 | 0.42 (0.18, 0.96) | | 0.041 |
| *Combined* | 1032 | 1.1% | 0.32 (0.15, 0.68) | | 0.006 | 0.24 (0.10, 0.62) | | 0.006 |

CI=confidence interval;

^1^ adjusted for individual level variables of gender, age category, occupation, living in household registration place or not, distance from nearest TB clinic, education level, income category, smear result at start of treatment and cluster level variable of pre-randomisation strata (rural/urban).

^2^ doses missed based on the larger of missed doses (1) using pill count, or (2) from the number of failures to open medication monitor.
