# Supplementary material for: Effectiveness of Electronic Reminders to Improve Medication Adherence in Tuberculosis Patients: A Cluster-Randomised Trial
Source: PLoS Med. 2015 Sep 15;12(9):e1001876. doi: 10.1371/journal.pmed.1001876 (PMC4570796; doi:10.1371/journal.pmed.1001876)
Supplement: S5 Table — (DOCX) [file pmed.1001876.s005.docx]

**S5 Table. Sensitivity analysis of the primary endpoint of the percentage of months with at least 20% doses missed (post-hoc sub-group analysis)**

|  | **Number of patients (row %)** | **Geometric mean of cluster level endpoint**^1^ | **Adjusted analysis**^2^ | | | **Number of patients (row %)** | **Geometric mean of cluster level endpoint** ^1^ | **Adjusted analysis**^2^ | | | **p-value for effect modification** |
| --- | --- | --- | --- | --- | --- | --- | --- | --- | --- | --- | --- |
|  |  |  | **Mean ratio**  **(95% CI)** | **p-value** | |  |  | **Mean ratio**  **(95% CI)** | **p-value** | |  |
|  | **No medication monitor problem^3^** | | | | | **Any medication monitor problem^3^** | | | | |  |
| *Control* | 895 (82.0%) | 27.3% | 1 | |  | 196 (18.0%) | 40.0% | 1 | |  |  |
| *Text messaging* | 829 (83.2%) | 24.8% | 0.93 (0.70, 1.24) | | 0.617 | 167 (16.8%) | 41.7% | 1.04 (0.77, 1.40) | | 0.777 | 0.449 |
| *Medication monitor* | 500 (50.4%) | 9.4% | 0.35 (0.18, 0.70) | | 0.006 | 492 (49.6%) | 23.9% | 0.61 (0.46, 0.80) | | 0.001 | 0.096 |
| *Combined* | 548 (51.7%) | 12.6% | 0.48 (0.24, 0.98) | | 0.045 | 511 (48.3%) | 16.0% | 0.42 (0.24, 0.74) | | 0.006 | 0.554 |

CI=confidence interval;

^1^of the percentage of months with at least 20% doses missed

^2^adjusted for individual level variables of gender, age category, occupation, living in household registration place or not, distance from nearest TB clinic, education level, income, smear status at start of treatment and cluster level variable of pre-randomisation strata (rural/urban).

^3^any medication monitor problem defined as *either* doctor reported any medication monitor problem *and/or* an incorrect date was recorded by the medication monitor, indicating the power had failed and then been resolved without resetting the internal clock to the correct date.
