# Supplementary material for: Effectiveness of Electronic Reminders to Improve Medication Adherence in Tuberculosis Patients: A Cluster-Randomised Trial
Source: PLoS Med. 2015 Sep 15;12(9):e1001876. doi: 10.1371/journal.pmed.1001876 (PMC4570796; doi:10.1371/journal.pmed.1001876)
Supplement: S6 Table — (DOCX) [file pmed.1001876.s006.docx]

**S6 Table. Problems with medication monitors and mobile phones by study arm**

|  | **Control** | | **Text messaging** | | **Medication monitor** | | **Combined** | |
| --- | --- | --- | --- | --- | --- | --- | --- | --- |
|  | **(n=1,104)** | | **(n=1,008)** | | **(n=997)** | | **(n=1,064)** | |
|  | **Num** | **%** ^1^ | **Num** | **%** ^1^ | **Num** | **%** ^1^ | **Num** | **%** ^1^ |
| **Medication monitor problems reported by doctor** |  |  |  |  |  |  |  |  |
| Number of problems | 108 |  | 116 |  | 821 |  | 789 |  |
| Cause of problems |  |  |  |  |  |  |  |  |
| Doctor set incorrectly | 0 | 0.0% | 3 | 2.6% | 1 | 0.1% | 2 | 0.3% |
| Patient used incorrectly | 0 | 0.0% | 5 | 4.3% | 238 | 29.0% | 191 | 24.2% |
| Medication monitor failure | 65 | 60.2% | 53 | 45.7% | 250 | 30.5% | 190 | 24.1% |
| No power | 20 | 18.5% | 31 | 26.7% | 214 | 26.1% | 323 | 40.9% |
| Other | 23 | 21.3% | 24 | 20.7% | 118 | 14.4% | 83 | 10.5% |
| Problem resolved |  |  |  |  |  |  |  |  |
| No | 21^#^ | 20.4% | 26 | 22.4% | 115^#^ | 14.1% | 65 | 8.2% |
| Yes | 82 | 79.6% | 90 | 77.6% | 701 | 85.9% | 724 | 91.8% |
| Medication monitor replaced | 62 | 57.4% | 48 | 41.4% | 172 | 21.0% | 112 | 14.2% |
|  |  |  |  |  |  |  |  |  |
| **Mobile phone problems reported by doctor** |  |  |  |  |  |  |  |  |
| Number of problems |  |  | 1476 |  |  |  | 574 |  |
| Cause of problems |  |  |  |  |  |  |  |  |
| Patient used incorrectly |  |  | 692 | 46.9% |  |  | 168 | 29.3% |
| Network failure |  |  | 287 | 19.4% |  |  | 146 | 25.4% |
| No money |  |  | 185 | 12.5% |  |  | 120 | 20.9% |
| Other |  |  | 312 | 21.1% |  |  | 140 | 24.4% |
| Problem resolved |  |  |  |  |  |  |  |  |
| No |  |  | 139 | 9.5% |  |  | 73 | 12.7% |
| Yes |  |  | 1331^$^ | 90.5% |  |  | 501 | 87.3% |

^1^ percentage denominator is number of problems in arm. Staff at the county level would resolve any problems and if necessary the medication monitor would be replaced.

^#^ these data were missing for five people in this arm

^$^ these data were missing for six people in this arm
