# Supplementary material for: Effectiveness of Electronic Reminders to Improve Medication Adherence in Tuberculosis Patients: A Cluster-Randomised Trial
Source: PLoS Med. 2015 Sep 15;12(9):e1001876. doi: 10.1371/journal.pmed.1001876 (PMC4570796; doi:10.1371/journal.pmed.1001876)
Supplement: S1 Text — (DOCX) [file pmed.1001876.s007.docx]

**S1 Text. Protocol**

**Sub-program of Patients’ Management of the China-Gates Foundation TB Project –**

Cluster Randomized Trial Protocol of using Mobile Text Messaging and Medication Monitor in TB case management

# Version 2.0; 10 May 2011

# Add trial registration number (when known)

#

Executive summary

1. Background

China has the second largest number of TB (Tuberculosis) cases in the world according to WHO’s estimate in 2010, next only to India. Finding and curing TB patients is a big concern for all those High TB burden countries. Directly Observed Treatment Short course (DOTS) strategy was testified the most effective TB case-management program so far and been implemented in China since 2001. Although great achievements have been made, the validity, accessibility and sustainability of DOTS are still being challenged, especially in west regions with poor economic development, traffic conditions, and shortage of medical and health resources. So WHO strongly recommends communities to provide Self Administration Treatment (SAT) methods for TB control. Thus, a new TB control method should be explored to address the deficiencies of DOT, which can not only ensure patient adherence but also accelerate limited health resources in playing greater roles. High developed new technology such as medication monitoring, mobile phone provided a good opportunity for TB case management. Patients could receive drug intake reminded-SMS and the behavior of taken drugs could be recorded by medication monitoring. Drug taken record probably give a Community health provider chance to study factors related to poor adherence and finally improve treatment adherence through changeable factors. But until now, there are no reported documents on TB case management by mobile phone technology or/and medication monitoring.

1. Aim

The study aims to explore and demonstrate innovative approaches for enhancing TB patient treatment management and establish improved practical programs for it.

1. Primary objective

To detect a 40% reduction in the mean proportion of months an individual has at least 3 doses missed in each intervention arm versus control arm.

1. Secondary objectives

- To detect reduction in adherence in each of the three intervention arms versus control arm including severe doses missing, overall doses taking,
- To describe TB treatment outcome in the control arm versus each of the three intervention arms.
- To describe satisfaction and feasibility of three intervention arms and to evaluate the cost-effect of this program.

1. Methods

The method of Cluster Randomized Trial (CRT) is used in this study. 36 research sites (counties /districts) are to be selected which are then randomized into 4 arms, i.e. 3 intervention arms and 1 control arm. Intervention arms will carry out management of initial treatment cases using mobile phone technology only, medication monitor only, and both mobile phone technology and medication monitor while control arm will exercise according to National TB Control Program.

After six month pilot study, 116 newly registered active TB patients (smear-positive or smear-negative) are to be recruited in each county (district) during the CRT and will receive 6 months anti-TB treatment. During case management period, information of the study will be collected, including the TB patients’ management records of taking drugs for the entire treatment (6 months), TB patients’ acceptability and satisfaction in each arm, case management cost of each arm.

TB treatment adherence will be compared between intervention and control arms. Based on all the information, new TB case management model for certain TB population will be developed.

1. Significance and transforming potential

The study will demonstrate the potential of using new tools to improve the adherence of TB patients’ behavior of taking drugs. If it is successful, the new case management model will be adopted by China National TB Program and the results will be published on international journals for information sharing.

| Name | Function | Telephone | E-mail |
| --- | --- | --- | --- |
| Shiwen Jiang | Director of the Trial (Design and Implement) | 8610 58900515 | jiangsw@chinatb.org |
| Lixia Wang | Coordinator of the trail | 8610 58900517 | wanglx@chinatb.org |
| Shiming Cheng | Coordinator of the trail | 8610 58900516 | smcheng@chinatb.org |
| Hui Zhang | Coordinator of the trail | 8610 58900514 | zhanghui@chinatb.org |
| Xiaoqiu Liu | Key staff to design and implement the trial | 8610 58900535 | leon@chinatb.org |
| Junying Chi | Project manager | 8610 63381073 | chijunying@chinatb.org |
| Xin Du | Surveillance of the trial | 8610 58900536 | stat@chinatb.org |
| Xue Li | Field visit for quality control and Data analysis | 8610 58900557 | lixue@chinatb.org |
| Jun Li | Field visit for quality control and Data analysis | 8610 58900535 | lijun@chinatb.org |
| Haibo Xie | Field visit for quality control | 8610 58900515 | xiehaibo@chinatb.org |
| Xiaoliang Yang | Field visit for quality control | 8610 58900535 | yangxiaoliang@chinatb.org |
| Hongguang Chen | Staff of management team (Finance, organization, supervision) | 8610 63381073 | chenhongguang@chinatb.org |
| Rong chen | Staff of management team (Analysis, monitoring) | 8610 63381073 | chenrong@chinatb.org |
| Mingming Liu | Staff of management team (Analysis, monitoring) | 8610 63381073 | liumingming@chinatb.org |

Study name

STUDY LEADERSHIP

COLLABORATING INSTITUTIONS

**Name of institution**

| Name | Function | Telephone | E-mail |
| --- | --- | --- | --- |
| Tuberculosis Dispensary, of Heilongjiang province | Collaboration Organization | 86 (451) 5515 7866 | hj@hl.jwst.gov.cn |
| Tuberculosis Dispensary, of Chongqing province | Collaboration Organization | 86 13650583933 | zs52077@sina.com |
| Tuberculosis Dispensary of Hunan province | Collaboration Organization | 86 13975185785 | [yhl4515@126.com](mailto:yhl4515@126.com) |
| Chronic and Epidemic center, Jiangsu Provincial Center for Disease Prevention and Control | Collaboration Organization | 86 13813914521 | [weiluxx@163.com](mailto:weiluxx@163.com) |
| London School of Hygiene and Tropical Medicine | Collaboration Organization | 44 20 7927 2889 | [Katherine.fielding@lshtm.ac.uk](mailto:Katherine.fielding@lshtm.ac.uk)  James.lewis@lshtm.ac.uk |

SPONSORS

**Add funder here**

| Name | Function | Telephone | E-mail |
| --- | --- | --- | --- |
| Daniel Chin | Sponsor from Bill & Melinda Gates Foundation | 86 10 84547500 | Daniel.chin@gatesfoundation.org |
| Shitong Huan |  | 8610 84547500 | Shitong.huan@gatesfoundation.org |
| Xia Liu | Sponsor from Chinese Ministry of Health | 86 10 68792556 | liuxia@moh.gov.cn |

**1. Background**

The global TB epidemic is growing increasingly serious, due to poverty, population growth, existing HIV/AIDS epidemics, as well as the poor effects of weak TB control programs ^[1]^. China ranks second in the world in number of TB patients, next only to India^［2］^. The WHO declared a global TB control emergency in April 1993^［3］^ and the solution to this situation was a global Directly Observed Treatment Short course (DOTS) strategy. A key point of this strategy is Directly Observed Treatment (DOT), which is designed specifically for strengthening treatment adherence. The WHO defines DOT as six to eight-months’ worth of regular treatment for TB patients who have already been found to be infectious, along with direct observation of patients’ drug intakes every time during the intensive phase, at the very least ^[4]^. This has been the most effective TB case-management program so far^［5］^.

1.1 The need to implement DOT

In accordance with the regulations of *Guidelines for the Planning and Implementation of the Prevention and Treatment of TB in China (2008)*, regular treatment implies that the actual number of times of drug intake should account for more than 90% of the total specified ones, during the specified period^［6］^. According to long-term scientific research and a large amount of treatment results, regular treatment may cure more than 95% of the patients with only 3% treatment failure (i.e. continual bacteria discharge). Irregular treatment mainly includes inconsistent (interrupted and/or discontinuous) drug intake and an unfinished specified course of treatment (stopping treatment ahead of time). Additionally, an irrational chemotherapy solution (e.g. a drug combination has not been used) is also a type of irregular treatment. Only 45% of patients have been cured by irregular treatment and around 50% of this type of treatment fails. A few serious cases may even end in death. Irregular treatment has a high failure and relapse rate, and in the meantime, is also very likely to cause drug resistance in bacteria to TB drugs, thus becoming a source of chronic infection ^[7]^.

In addition, current scholars at home and abroad are nearly undivided in the conclusion that TB existing in nature contains an extremely small amount of drug-resistant bacteria. The chance of drug resistance to a certain TB drug due to chromosomes’ spontaneous mutations is extremely low. Drug-resistant TB is caused by human factors such as irregular treatment and unreasonable drug intake. Retreatment of drug-resistant TB patients does not only result in poor effects but also transmits drug-resistant bacteria, which is a major threat to an individual’s health as well as a serious public health problem.

1.2 Status of DOT implementation in China

It is stipulated in *Guidelines for the Planning and Implementation of the Prevention and Treatment of TB in China (2008)* that smear-positive TB patients should exercise full-time monitoring chemotherapy with direct observation from medical staff. Village/community doctors, as well as medical staffs of enterprises and government institutions, are responsible for monitoring chemotherapy ^[8]^. Taking into consideration low feasibility of medical staff monitoring, *Guidelines* also states that active pulmonary TB patients are all objects of treatment and management, among whom smear-positive TB patients are key. In principle, patients’ treatment management is monitored by medical staff, but if patients live more than 1.5 kilometers away from the village health clinic (community health service station) or village/community doctors cannot carry out the monitoring task, family member or volunteer monitoring can thus be practiced^［6］^.

1.3 Limitations of DOT implementation

Many countries in the world have contributed to the huge success of TB control in recent years, with implementation of the DOTS strategy. What is beyond dispute is that factors such as free medication supply and government support and commitment have indeed accelerated TB control work, whereas whether or not DOT is absolutely necessary is an ongoing debate among researchers all over the world^[12]^.

1.3.1 Validity of DOT is being challenged

Although DOT has become the best case management method recommended by the WHO and great achievements have been made in certain countries and regions, the validity of DOT is still being challenged. Statistics^［15］^ show that among the 10 trials which have been carried out, DOT is not necessarily more effective than Self Administration Treatment (SAT). It is also stated in some research that other interventions are still needed to ensure the 85% treatment completion rate ^[16~18]^.

1.3.2 DOT is unsuitable to all districts and groups

Many problems have also occurred during the implementation of DOT in China. It is difficult to implement the whole course of treatment (six to eight months), especially in the western regions with poor economic development, traffic conditions, and medical and health services, and among people who’s working hours and drug intake times conflict with each other. According to research by Hu Daiyu, etc. on 401 smear-positive TB cases in four counties (prefectures) in Chongqing, only 64 patients (15.96%) exercised DOT, while there were as many as 289 patients (72.07%) that were not monitored for drug intake.

Furthermore, DOT is not necessary for all patients. A research done on 122 SAT TB patients in America showed that after patients removed isoniazid from the non-medication monitoring and took masalazine for 18 to 24 months, 82.3% of them realized 70% of their total specified pill burden and 60.7% of them have even exceeded 90% of this number^［20］^. This indicates that patients with high treatment adherence can practice SAT, whereas auxiliary measures should be taken to ensure treatment adherence on those with poor adherence.

1.3.3 Implementing DOTS strategy on all TB patients has increased a heavy burden on health system as well as patients themselves

China ranks second in the world in number of TB cases, next only to India^［21］^, which decides great demand for supervisors. Due to limited health resources, it is a huge burden to carry out DOT management on all TB patients several times a week. It is impossible for those who live far away or who have conflicting working hours to exercise DOT with medical staff. For example, research in some areas show that monitoring rates of medical staff are relatively low. In remote areas, family members become primary choices for supervisors^［24］^.In the widespread rural areas, community members are busy with farm work and do not take the time to monitor medication intake, and thus full-course monitoring turns out to be a mere formality. Additionally, it is still difficult to carry out volunteer monitoring in China. As a result, what is actually exercised in China is full-course management or self drug-intake, which may not ensure consistent drug intake and a high cure rate.

Lienhardr and Ogden point out that some factors concerning TB control are unsustainable, e.g. patients’ different attitudes towards the disease, extremist patients’ care changes, high treatment fees shouldered by patients, increased social discrimination, non-implementation of DOTS strategy, etc. as they all need financial support. Therefore, general application of DOT is being challenged. In addition, a similar result is shown in a randomized clinical trial (RCT) with drug resistance^［26］.^Khan and others also point out that the cost benefit of DOT is lower than that of SAT.

1.3.4 Acceptability of DOT

TB patients’ attitudes towards DOT vary. Research shows that most patients consider it to be unnecessary because they insist on taking medication spontaneously, and many want to be cured as soon as possible, so they don’t think monitoring from doctors is needed. There are also some patients who consider it to be inconvenient, as it influences both their doctors’ work, as well as their own working hours. There are, however, some individual patients willing to carry out DOT, as they consider it to be the doctor’s responsibility; they also feel that it shows their physicians’ care for them, which is helpful for patients older in age and patients who have poor drug adherence to complete the entire course of treatment^［28］^. With this in mind, case management should therefore be adapted to patients’ varying attitudes.

1.3.5 Drug adherence problems still exist in the implementation process of DOT

Adherence refers to the degree of conformity between a patient’s behavior and a doctor’s prescription in treatment and prevention of the disease. People began to know more about TB patients’ treatment adherence starting in the 1950s.

Since the treatment course recommended by standard TB treatment programs is a “long-term” process for most patients, their treatment adherence has become a key factor in TB control and has attracted more and more attention. Although governments have made constant improvements in TB control and prevention, treatment adherence is still unsatisfactory due to certain social and personal factors, and the proportion of irregular treatment is still high ^[32-33]^. According to research done by Zheng Yuqun on 670 TB patients in Jiangsu province, 82 of them (12.2%^［34］^) were found to be taking medication irregularly during treatment. A research done on 404 Ethiopian smear-positive patients found that 81 (20%) of them quit and 91% were interrupted during treatment duration^［35］^. It was also found in the 26 weeks of follow-up study done on 300 new TB cases in Cambodia that 65.6% of them did not take medication according to instructions during the entire course^［36］^.

Many scholars have conducted surveys on influencing factors of TB patients’ drug adherence, which mainly include the following: firstly, patients’ factors such as gender, age, educational level, and knowledge of TB, etc.; secondly, medication factors which mainly affect patients’ drug adherence such as adverse reactions to TB drugs, duration of treatment, etc.; thirdly, economic factors: research shows that most patients regularly taking medications from hospitals are motivated by transportation compensation and liver protection medication compensation ^[42]^. However, there is also research showing a high irregular drug intake rate among those who enjoy excessively high income; fourthly, social factors such as social support, including support from family members, neighbors, relatives and doctor-patient relationship, etc.; fifthly, the seriousness of patients’ illnesses also has an impact on adherence. Research shows that those who are not seriously ill have relatively full confidence in being cured, as well as good drug adherence, whereas those who are more seriously ill tend to lose confidence, further leading to poor drug adherence^[44]^.

1.4 How to address weaknesses of DOT implementation

In order to address problems faced by many health systems offering DOT to TB patients, it is no longer insisted by the new international standard that all patients should exercise DOT, yet it is also clearly defined that medical staff should not only conduct correct treatment programs, but also assess patients’ treatment adherence and take immediate actions upon recognition of irregular drug intake^［49］^. The WHO strongly recommends communities to take part in TB control and provide monitored SAT methods. Community workers may find out about patients’ treatment adherence through monitoring records and focus on those with poor adherence. In this way, fewer supervisors will be needed, resolving issues of overstaffing. Thus, a new TB control method should be explored to address the deficiencies of DOT, which can not only ensure patient adherence but also accelerate limited health resources in playing greater roles.

1.4.1 Medication monitoring

The medication monitor, which mainly consists of a container of medications and interior devices for monitoring drug intake, has recently been developed by some countries. It reminds patients to take medications on time. For SAT patients, medical staff will put a certain amount of medicationn into the medication monitor, and every drug intake will be recorded by the interior device. Though medication monitors cannot actually prove that drugs removed by patients have actually been ingested, this method can assess patients’ treatment adherence more accurately if compared with traditional methods; meanwhile, the cost of medication monitor is relatively low and a medication monitor can be refilled with medications over and over again.

Many researchers have pointed out that medication monitors can record daily dosages and times of intake, yet a patient’s drug adherence tends to decline with the rise of dosage^［50］^. In the meantime, medication monitor technology has also been applied to follow-up evaluations of antiretroviral drug adherence in treating HIV/AIDS patients^［51-53］^. Furthermore, it has also been proven to be effective in health education and information feedback. The research done on 104 Canadian latent TB patients’ treatment adherence shows that treatment completion rate is closely related to the first month’s drug intake rate. Apart from this, it is more likely for those who take their medication at almost the same time every day to complete the treatment^［54］^. However, according to the test on Haitian active patients, the probability of better adherence of those with “more than 90% of adherence during the first 11 weeks” within one year is three times higher than that of those with “less than 90% of adherence during the first 11 weeks” and the treatment failure rate of those with “more than 90% of adherence during the first 11 weeks” is six times lower than that of those with “less than 90% of adherence during the first 11 weeks”. All this research has shown that early medication monitoring records can help to predict patients’ adherence and failure^［55］^.

The usage of medication monitors can predict patients’ adherence and identify those with better adherence. Research shows that at least 60-65% of patients have good adherence and can carry out self-management successfully. Resources saved from those who do not need DOT can be used for those with poor adherence. Additionally, guidance, direct observation, and extended treatment duration should be carried out when necessary, which can lighten the illness burden greatly.

1.4.2 Telephone

The telecommunication industry is developed rapidly in China. Home telephones have been installed in almost every family home in rural areas, and some also have mobile phones. Advanced telecommunication tools have provided a good opportunity for TB case management. Nan county of Hunan province carried out TB case management by using telecommunication tools, i.e. telephone monitoring of new smear-positive TB patients from January to June in 2008, and achieved favorable effects. Telephone monitoring can not only ensure high cure rates and regular treatment rates of new smear-positive TB patients, but it also has other advantages as well. Firstly, telephone monitoring helps to protect patient privacy, making this method more acceptable to them. Secondly, it is both convenient and easy. As well, it increases monitoring frequency. For example, county-level monitoring increased from two follow-ups to 12 telephone monitors, which not only made it easier for county-level doctors to find out about side effects of the drugs used, but also made it easier for them to deal with these problems in a timely manner. They also get to know about the actual implementation of country/village/community doctors’ monitoring. Finally, telephone monitoring costs less; thus, saving a large amount of money and time for other work concerns TB control^［56］^.

Similarly, mobile phone technology also has the potential to help improve TB case management, and it is easy to monitor and assess TB control work. Taking into account the vast number of mobile phone users, we believe that there will be a bright future if this technology is used to improve public health services. Currently, there are no reported documents on TB case management by mobile phone technology.

1.4.3 The method to set up reminders

Setting up reminders means offering detailed medication dosages, using methods of drug intake and placing cards on places where patients can see them, e.g. on a bedside table, dining table, or near the toothbrush holder, etc., which is one of the good practices in increasing patients’ adherence. According to Sonnenberg^［57］^ and others’ research, after cards with drug intake instructions were offered to patients, their drug adherence increased from 62.0% to 88.0-93.0%. There is also research reporting that reminding patients by sending letters to them twice when they forget to take drugs from the hospital also results in better adherence ^[58]^. Other related research shows that among 29 patients of the variable group who stopped taking medications from the hospital, 17 (58.16 %) of them went back to the hospital to continue treatment after reminder letters were sent to them, while only 4 (12.19 %) out of the 31 patients of the control group went back without any measures taken^[59]^.

Overall, it is necessary to explore new technologies and methods for TB case management in order to improve TB patients’ treatment adherence and ensure their completion of their specified course of treatment.

**2. Aims and objectives of the research**

2.1 Aims of the study

The study aims to explore and demonstrate innovative approaches for enhancing TB patient treatment management and establish improved practical programs for it.

Specific aims of the study

2.1.1 To establish TB case management procedures and technological plan using innovative mobile phone technology, medication monitor and the combination of the two technologies and develop incentive structures for health care providers who will implement the TB case management..

2.1.2 To compare the TB patient treatment adherence, treatment outcomes and service-receiving population between current NTP case management program and the 3 new models (mobile phone model, medication monitor model and the combination of the two technologies model)

2.1.3 To compare the feasibility and acceptability between current NTP case management program and three new models.

2.1.4 To analyze and assess the current NTP case management program and three new models in terms of their cost effectiveness.

2.2 Primary objective

To detect a 40% reduction in the mean proportion of months an individual has at least 3 doses missed in each intervention arm versus control arm.

2.3 Secondary objectives

Secondary I objectives - adherence

- To detect a 40% reduction in the mean proportion of months with at least 7 doses missed in each of the three intervention arms versus control arm.
- To detect a 40% reduction in the mean proportion of overall missed doses in each of the three intervention arms versus control arm.
- To detect a 40% reduction in the proportion non-adherent (as defined by the NTP; at least 10% of doses missed) in each of the three intervention arms versus control arm.

Secondary II objectives - treatment outcomes

- To describe the proportion of patients defaulting during TB treatment (as defined by the NTP; being lost for at least 2 months during the TB treatment period) in the control arm versus each of the three intervention arms.
- To describe the proportion of smear positive TB cases who become smear negative at 2 months in the control arm and each of the three intervention arms.
- To describe the proportion of patients with treatment outcome of cure or completed treatment in the control arm and each of the three intervention arms.

Secondary III objectives - satisfaction and feasibility

- To describe difference in the mean satisfaction score in the control arm versus each of the three intervention arms.
- To describe the feasibility of the interventions with respect to the patients, health care providers and TB control programme point of view.

Secondary IV objectives - cost

- To describe the costs of the three interventions and the cost effectiveness of the three interventions versus control.

**3. Research design**

The method of Cluster Randomized Trial (CRT) is used in this study. 36 research sites (counties /districts) are to be selected which are then randomized into 4 arms, i.e. 3 intervention arms and 1 control arm. Intervention arms will carry out management of initial treatment cases using mobile phone technology only, medication monitor only, and both mobile phone technology and medication monitor while control arm will exercise according to National TB Control Program.

Information of the study will be collected, including the TB patients’ management records, of taking drugs for the entire treatment (6 months), TB patients’ acceptability and satisfaction in each arm, case management cost of each arm. TB treatment adherence will be compared between intervention and control arms. Based on all the information, new TB case management model for certain TB population will be developed.

The CRT study will begin in June, 2011 and the duration is 15 months. This follows a piloting phase from November 2010-May2011. Case enrolment will start at the begin in June 2011 and the enrolment duration is 6 months. Once the TB patient is enrolled, he/she will receive 6 months’ regular anti-TB treatment besides all the interventions of a certain arm he/she is designated to. During the treatment, county level researchers will carry out follow up on a monthly basis.

Detailed flow chart of the study is as follows:

**4. Selection of clusters**

36 counties (districts) in Chongqing, Hubei, Jiangsu and Heilongjiang provinces will be selected for the CRT study, each county (district) as a cluster.

4.1 Selection Criteria

4.1.1 The registration of active pulmonary TB patients in the county (district) is more than 300 cases in 2009 (including 300 cases);

4.1.2 Nine counties (districts) are selected from each province, the ratio of county and district is 1:2. Except Chongqing (Chongqing is a province level municipality), every province is required to select nine counties (districts) with the ratio of 4 counties (districts) in one city and the 5 counties (districts) in the other; and each city has at least one district selected.
 4.1.3 The counties (districts) selected should be capable of participating in the study and be willing to do so.
 Note: Definition of the county (district)
 District: non-agricultural population in total area accounts for more than 50% of the population (including 50%).
 County: non-agricultural population in total area accounts for less than 50% of the population.
 4.2 Selection Process
 4.2.1National implementation agencies firstly develop a list of counties (districts) with more than 300 active TB patients annual registration according to the registration of 2009 in the 4 provinces and exclude those failing to meet the requirements.

4.2.2 Excluding Chongqing, two cities were chosen in the project provinces under the national-provided list, and each have at least four counties (districts) meet the standards.

4.2.3 Excluding Chongqing, 9 counties (districts) which have the ability and willingness to participate in the study were chosen in each project province. 4 counties (districts) will be chosen in one city, and 5 counties (districts) in the other city, and each has at least one district.
 4.2.4 Nine counties (districts) which are capable and willing to participate in the study were chosen according to the list of counties and according the ratio of counties and districts.

4.2.5 Chongqing simply selected nine counties (districts) which are capable and willing to participate in the study from the national-provided list according the ratio of counties and districts.

4.2.6 The list of selected counties (districts) is required to be reported to national implementation agency.

**5. Selection of Participants**

116 newly registered active TB patients (smear-positive or smear-negative, patients excluding patients of simple tuberculosis pleurisy and non sputum smear tested) are to be recruited in each county (district) during the CRT

Enrolment criteria for the patients of the study:

- - - Be willing to participate in the study;
    - conscious without any mental disease；
    - conscious without any visual, auditory or language impairment；
    - At least 18 years old；
    - Patient or family member is able to read SMS and use medication monitor after training.

**6. Intervention Measures**

There are four arms in the trial, i.e. three intervention arms and one control arm. The specific intervention measures for each arm are as follows.

6.1 Mobile Phone Arm

Providing participants (patients) with mobile phones as reminding tools and equipping them with medication monitor without reminding function to record the patients’ drugs intake; when participants receiving SMS of reminding taking drugs, they should take drugs immediately and make feedback by SMS. Doctors would not have access to data from the medication monitor. Doctors in TB dispensary would collect the SMS feedback from patients to assess how many doses are missed in a month. Based on the missed doses, additional intervention and incentive mechanism should be implemented which is described as following:

If missed doses are 3-6 doses within a month, township doctors should visit patient twice a month, village/community doctors should visit patient once per week and the other treatment management should not be changed. Township doctors would be incented with 10 Yuan (RMB) per visit, and village/community doctors would be incented with 5 Yuan (RMB) per visit.

If missed doses are more than 7 doses (including 7 doses) within a month, or the patients missed 3-6 doses once again, village/community doctors should adopt DOTS to supervise these patients to take drugs. Village/community doctors would be incented with 5 Yuan (RMB) per visit.

6.2 Medication Monitor Arm

Offering medication monitor with reminding functions to patients for reminding and recording drug intake; Doctors in TB dispensary would collect the drugs intake record from medication monitor once a month to assess that how many doses are missed in a month. Based on the missed doses, additional intervention and incentive mechanism should be implemented which is mentioned before in 6.1.

6.3 Mobile Phone and Medication Monitor Arm

Providing mobile phone and also medication monitor with reminding function for patients as tools for communication, reminding and recording drug intake; the drugs intake record from medication monitor and SMS from patients should be collected once a month. Doctors in TB dispensary would assess that how many doses are missed in a month through the drug intake record of medication monitor. Based on the missed doses, additional intervention and incentive mechanism should be implemented which is mentioned before in 6.1.

6.4 Control Arm

Managing the newly registered active TB patients with the initial treatment by the current national TB control program, and providing patients with medication monitor without reminding function; the medication monitor would be used to ensure recording drugs intake that can be compared with intervention arms. Doctors would not have access to data from the medication monitor.

**7. Data Collection and Monitoring**

All data for the trial is collected by four linked databases: enrolment, mobile phone management database (only for those patients in the mobile phone or mobile phone + medication monitor arm), medication monitor management database and patient’s management record respectively. All patients should provide his general information when being recruited (more detail is showed in Appendix 3). The information about medicine intake, use of new technology, result of smear examination at 2 months and treatment outcome will be kept in patient’s management record (more detail is showed in Appendix 4).

Every patient has a unique study number and this special number should be kept same in these four databases due to guaranteeing the reliability of the data. The medicine intake record exported from medication monitor each month cannot be revised by his supervisor or patient himself to avoid deliberate modification.

**8. Statistical considerations**

8.1 Endpoints

The endpoints will be measured in all individuals who consented and used the medication monitor for dispensing, regardless of whether they used the reminder functions of the medication monitor or the mobile phone technology. A per protocol analysis will be conducted for the primary adherence outcome and will be defined in advance of data analysis.

The endpoints are grouped into four categories: (i) adherence,(ii) treatment outcomes, (iii) satisfaction/feasibility, (iv) cost.

Primary endpoint

The primary endpoint is a quantitative measure of the proportion of months on TB treatment where at least 3 doses were missed in a month.

Secondary I endpoints - adherence

1. Proportion of months with at least 7 doses missed: This is a quantitative measure based on the proportion of months on TB treatment where at least 7 doses were missed in a month.
2. Proportion of overall missed doses: This is a quantitative measure based on the total number of missed doses over the TB treatment period.
3. Non-adherence (as defined by the NTP): This is a binary measure and is based on the definition as used by the NTP of at least 10% of doses missed.

Secondary II endpoints - treatment outcomes

1. Defaulting during TB treatment: This is a binary measure defined as being lost for at least 2 months during the TB treatment period.
2. Smear conversion at 2 months: This is a binary measure based on those patients smear positive at enrolment and defined as being smear negative at 2 months from start of TB treatment
3. Cure/completed treatment. This is a binary measure defined as being cured/completed treatment at the end of the TB treatment period.

Secondary III endpoints - satisfaction/feasibility

1. Acceptability/ satisfaction of intervention by patients
2. Feasibility will be explored in focus group discussions with patients, health care providers and TB control programmes.

Secondary IV endpoints - costs

1. Costs - Cost per patient managed: Cost for managing a patient for entire treatment using new approach or control approach

8.2 sample size

As there are no data to inform sample size calculations based on the primary adherence outcome the sample size calculations have been based on a binary outcome of “poor adherence”. Assuming 9 clusters per arm and 110 TB patients per cluster, a two-sided type I error of 0.05, a coefficient of variation in true proportions between clusters of 0.3 and the percentage with “poor adherence” in the control arm of 40% then the study will have 84% power to detect a 40% reduction in the outcome in the intervention arm (either mobile phone arm or medication monitor arm or combined mobile phone and medication monitor arm). Table 1 shows the power under various scenarios assuming 9 clusters per arm and 110 TB patients per cluster and a two-sided type I error of 0.05.

Table 1: Study power assuming 9 clusters per arm, 110 TB patients per cluster, a type I error of 0.05 and (i) the coefficient of variation ranging from 0.2 to 0.3; (ii) % with poor adherence in control arm ranging from 20-40%; (iii) % reduction in poor adherence in intervention arm ranging from 30-40%.

|  |  |  | Power for | | |
| --- | --- | --- | --- | --- | --- |
| % in  Control  arm | % in  intervention  arm | % reduction | k=0.30 | k=0.25 | k=0.20 |
| 40% | 24% | 40% | 84% | 93% | 98% |
| 30% | 18% |  | 81% | 90% | 96% |
| 20% | 12% |  | 75% | 84% | 91% |
|  |  |  |  |  |  |
| 40% | 26% | 35% | 71% | 83% | 93% |
| 30% | 19.5% |  | 68% | 79% | 89% |
| 20% | 13% |  | 61% | 72% | 82% |
|  |  |  |  |  |  |
| 40% | 28% | 30% | 55% | 69% | 83% |
| 30% | 21% |  | 52% | 64% | 77% |
| 20% | 14% |  | <50% | 56% | 67% |

k=coefficient of variation; Power calculations are based on a pairwise comparison of each intervention arm with the control arm (9 clusters vs 9 clusters; 110 patients per cluster).

The sample size per cluster has been adjusted to take into account 5% of medication monitors malfunctioning. Therefore 116 TB patients per cluster will be recruited into the study. Using data from the pre-trial phase and before recruitment ends during the main recruitment phase the percentage of medication monitors malfunctioning will be reviewed and if greater than 5% the sample size may be increased.

8.3 Preliminary plan for data analysis

The analysis plan is based on the primary and secondary outcomes for adherence (I).

Description of the intervention and control arms at baseline

The baseline characteristics of clusters (district/county) in the intervention and control arms will be described as follows:

The number of TB patients treated in last three years; TB outcomes in last three years; number of townships/villages; number of doctors delivering TB care; will be tabulated by intervention status (four groups) at the cluster level using the mean, median and range. Percentage of population defined as urban and routinely collected data on social, economic and demographic characteristics at the cluster level will be tabulated by intervention status at the cluster level using the mean, median and range. TB outcomes in last three years will also be tabulated by study arm, ignoring clusters, using frequencies and percentages.

Table x.1: sample table showing summary of data at baseline, at the cluster level

|  | | Control | Mobile phone only | Medication monitor only | Mobile phone and medication monitor |
| --- | --- | --- | --- | --- | --- |
| At cluster level | | (n=9) | (n=9) | (n=9) | (n=9) |
| # TB patients* | Median (range) | 470(411~768) | 478(304~1022) | 677(314~1332) | 487(359~757) |
| TB outcomes* |  |  |  |  |  |
|  | Smear Positive |  |  |  |  |
|  | Cured | 93.9(87.5~99.6) | 94.6(89.7~99.7) | 95.6(92.0~98.8) | 90.8(81.6~97.6) |
|  | Default | 0(0~0.88) | 0(0~1.45) | 0(0~1.95) | 0(0~1.36) |
| # doctors delivering TB care* | Median (range) | 11(9~38) | 12(4~19) | 11(6~32) | 12(5~17) |
| % of cluster rural | Median(range) | 66.5(9.3~87.4) | 61.1(0~87.0) | 70.4(4.0~86.2) | 72.9(20.2~83.9) |
| Other social, economic & demographic characteristics** | |  |  |  |  |
| Population(,000) | Median(range) | 650(406~1407) | 543(298~1242) | 897(702~1724) | 724(394~1013) |
| Per GDP(Yuan) | Median(range) | 20936(6600~41620) | 15623(6338~56763) | 16274(4978~64455) | 11530(6167~53973) |
| Area (Km^2^) | Median(range) | 1477(171~4616) | 1892(100~4026) | 1142(183~3634) | 2046(432~4702) |

* in 2009 ** from routine data

Description of TB patients recruited into the study the intervention and control arms at baseline

The baseline characteristics of TB patients recruited into the study in the intervention and control arms will be described as follows:

Socio-demographics (age, gender, ethnicity, language, marital status, employment status and category, education level; literacy, migrant/permanent, # people in household, health insurance, family income, mobile phone ownership); TB diagnosis variables (smear status; how diagnosed; clinical presentation [symptoms, weight loss; BMI], CXR reading); distance to dispensary; cost to visit dispensary; payment for additional tests/drugs; treatment supporter will be tabulated by intervention status (i) at the individual level (by study arm, ignoring clusters) and (ii) at the cluster level using the mean, median and range.

Table x.2: sample table showing summary of baseline data from patient enrolled into the study

|  |  | Control | Mobile phone only | Medication monitor only | Mobile phone and medication monitor |
| --- | --- | --- | --- | --- | --- |
| Ignoring clusters | | (n~990) | (n~990) | (n~990) | (n~990) |
| Age (years) | Median (range) |  |  |  |  |
| Sex | Female: n (%) |  |  |  |  |
| Ethnic group | Group A: n (%)  Group B: n (%)  Group C: n (%) |  |  |  |  |
| Etc. |  |  |  |  |  |
| At cluster level | | (n=9) | (n=9) | (n=9) | (n=9) |
| Age (years) | Median (range) of %  <40  40-50  ≥50 |  |  |  |  |
| Sex | Median (range) of % female |  |  |  |  |
| Ethnic group | Median (range) of % in  Group A  Group B  Group C |  |  |  |  |
| Etc. |  |  |  |  |  |

All characteristics will be compared by arm of the trial to ensure comparability. Any variables for which there is a substantial imbalance will be noted so that final analysis can take this into account (see section 5.8.3.6). This assessment will not be based on the results of significance tests, and p-values will not be shown.

Process measures of interventions in the intervention clusters

A summary of process measures for the mobile phone only; medication monitor only and mobile phone and medication monitor will be tabulated overall (ignoring cluster) and at the cluster level using the mean, median and range.

In the intervention arms using the mobile phone technology the percentage of patients who agree to use the mobile phone reminder system will be tabulated overall and by cluster using median and range. In the intervention arms using the medication monitor and/or mobile phone technology the percentage of patients who using the reminder functions will be tabulated overall and by cluster using median and range.

Imbalance of clusters in the intervention and control clusters

Potential confounding factors will be adjusted for. These will include

i) a priori: XXX which are known to be important determinants of many of the study outcomes.

ii) any other factors which are found to differ between the study arms at baseline and which may be a risk factor for the outcome (listed in section 8.3.1).

Per protocol and subgroup analyses

For the primary outcome, a secondary analysis will be conducted restricted to participants who satisfy the per protocol definition. This is most relevant to the intervention arms whereby participants who demonstrate adequate use of the intervention will form the analysis group. The per protocol classification will be defined in advance of data analysis.

For the primary outcome, subgroup analyses by the following factors will be performed:

Age group (2 groups; groups to be defined in advance of data analysis)

Literacy (low/high literacy)

Gender

Urban/rural

Statistical methods

The analysis is based on a total of 36 clusters. Three pairwise comparisons will be conducted: (i) mobile phone only versus control; (ii) medication monitor only versus control ; (iii) mobile phone and medication monitor versus control. Each pairwise comparison will be based on 18 clusters. Prior to randomisation clusters will be grouped into two strata, based on an urban/rural definition.

Summary measures

For the quantitative primary and secondary outcomes the overall mean, combining data across clusters, will be reported by intervention arm. For binary secondary outcomes the overall risk, combining data across clusters, will be reported by intervention arm.

Unadjusted analysis

The analysis will give each cluster equal weight. For quantitative outcomes the mean for each cluster will be calculated. For binary outcomes the overall risk for each cluster will be calculated. Mean/risks for each cluster will be shown, by strata and arm.

For quantitative outcomes the mean difference and associated 95% CI for each arm of the study will be reported. The mean difference will be estimated using linear regression of the mean outcome on stratum and arm. An approximate standard error for the difference in mean outcome between arms is obtained based on the residual mean square from a two-way analysis of variance (ANOVA) of the cluster mean outcome on stratum, study arm and the interaction between stratum and study arm. The 95% CI is calculated from this standard error, using a t-statistic with 14 degrees of freedom (for a pairwise comparison of two arms).

For binary outcomes a log transformation (where necessary) will be applied to the risk for each cluster. The mean and SD of these log risks will be used to obtain the geometric mean (GM) and associated 95% CI for each arm of the study. An approximate standard error for the difference in log (GM) between arms is obtained based on the residual mean square from a two-way ANOVA of the cluster log-risk on stratum, study arm and the interaction between stratum and study arm.

The 95% CI is calculated from this standard error, using a t-statistic with 14 degrees of freedom.

Adjusted analysis

Factors for adjustment will be determined as stated above.

Depending on the outcome to be analysed, linear or logistic regression will be used to adjust for confounders at the individual level and cluster level, adopting a two-stage approach.

The regression model will include terms for the adjustment factors and strata, but not study arm. For binary outcomes: for each cluster the fitted model will be used to obtain the ratio of observed to expected (O/E) events, and a log transformation will be applied to this ratio, where appropriate. Linear regression of the log (O/E) on stratum and arm (2-way ANOVA) will be used to estimate the risk ratio. The variance for the ratio of mean O/E is calculated from the residual mean square from an ANOVA of O/E on stratum, arm and the interaction between stratum and arm. The 95% CI is calculated from this variance, using a t-statistic with 14 degrees of freedom.

Supplementary analyses

Supplementary analyses may include an analysis of risk factors for poor adherence among individuals enrolled into the study using information on exposure variables collected at enrolment.

**9. Ethical Issues**

This research will apply for the permission of morals to the Committee for Morals in Scientific Research in London School of Hygiene & Tropical Medicine and Chinese Centre for Disease Control and Prevention.

When selecting research objects, it is necessary to inform each research participant of relevant information and specific contents in detail as well as the methods being adopted in the research, i.e. mobile phone, medication monitor as well as mobile phone and medication monitor, to manage the TB treatment. In addition, it should be noted clearly that if the participants participant in the research, they would benefit from it, for the doctor could acquire the performance of patients’ TB treatment earlier so as to intensify the patients’ management and ensure earlier recovery. For this research does not involve the change of drugs, treatment plans and so on, the participant will not run any risk resulting from taking part in this research. The researcher is required to make a statement to the participant: The participant is entirely willing to take part in the research; the participant could withdraw from the research at any time in the research without any punishment or unfavorable treatment; all information collected from the participant in the research is confidential and only confined to the use of the researcher. Any private information including name and contacts and so on can not be published in any journals, newspapers and so on. The informed consent with the researchers should be signed with all the participants’ consent and on the premise of participants’ willing.

**10. Timeline**

See as follows

Form 2 Research Schedule

| Contents | Major activities | Starting time | Concluding time |
| --- | --- | --- | --- |
| Draw up implementation plan of community trials | Convene seminar(discuss community trials approaches) | August 3rd, 2009 | August 5th, 2009 |
|  | Draw up community trials implementation plan | December 31st, 2009 | January 1st, 2010 |
|  | Convene seminar,(sample, Selecting pilots) | January 4th, 2010 | January 8th, 2010 |
|  | Convene seminar,(trials design and tools for data collection) | January 14th, 2010 | January 16th, 2010 |
|  | Convene trial experiment in 6 counties (districts) | January 10th, 2010 | March 31st, 2010 |
|  | revise community trial implementation plans and implementation details | April 19th, 2010 | April 21st, 2010 |
|  | Medication monitoring and software development and testing | May,2010 | July, 2010 |
| Training for community trials implementation plan | The purchase and distribution of medication monitor and mobile phone | August,2010 | September,2010 |
|  | Central implementation agencies’ training on provincial and municipal implementation agencies | October,2010 | October,2010 |
|  | County implementation agencies’ training on township and village implementation agencies | October,2010 | October,2010 |
| Trial operation of community trials | Conduct test on community trials in all counties (districts) | November 1st,2010 | May 30th,2011 |
| Conduct the community trials implementation | The research objectives(TB patients) in line with the requirements | June 1st,2011 | December 31st, 2011 |
|  | All research arms conduct intervention on patients, follow-up visits and information collection | June 1st,2011 | May 31th, 2012 |
| Summarize the community trials | Data collection, compiling and analysis | May 31th,2012 | June 30th,2012 |
|  | Conduct feasibility or cost benefit evaluation on community trials | May 31th,2012 | June 30th,2012 |
|  | Draw up research report | July 1st, 2012 | September 30^th^, 2012 |

**11. The Policy for Reporting, Dissemination and Publication**

11.1 Publication of Articles

The articles summarize the experiment design of employing mobile phones and medication monitors to conduct management of TB patients as well as the experience and methods in the implementation process. Articles below will be published after the project:

| Titles of articles to be published | journals to publish articles |
| --- | --- |
| The Research on Methods of Mobile Phone Management for TB Patients | *Chinese Journal of Antituberculosis, Chinese Journal of Tuberculosis and Respiratory Diseases, Chinese Journal of Epidemiology, International Journal of Epidemiology and Infectious Disease, International Journal of Tuberculosis and Lung Disease, China Journal of Public Health, LANCET* |
| The Research on Methods of Medication Monitor Management for TB Patients |  |
| The Research on Methods of Mobile Phones and Medication Monitor Management for TB Patients |  |
| The Research on Adherence Analysis of TB Patients’ New Treatment Mode |  |
| The Research on Cost Benefit of TB Patients’ New Treatment Mode |  |
| Progress of TB Patients’ New Treatment Mode |  |
| Research on Detecting Drugs Intake through Urine Examination in TB Patients’ New Treatment Mode |  |

11.2 Dissemination and Application of Research Findings

11.2.1 National Relevant Meetings

Opportunities as meetings of higher level for TB prevention and treatment, national meetings of annual work for TB prevention and treatment, academic meetings of professional committee of TB control in China’s Antituberculosis Association should be taken to introduce the achievements and experience acquired in employing new methods for TB patients’ management.

11.2.2 International Relevant Meetings

Opportunities as international relevant meetings should be taken to introduce the achievements and experience acquired in employing new methods for TB patients’ management.

11.2.3 Domestic Development

The research findings in patients’ new management methods should be summarized so as to be spread to 200 counties (districts) for demonstration in the second phase of the project. If the second phase of the project succeeds, it will be spread to other regions in the nation.

11.3 Monograph Publication

Monograph named “TB Patients’ New Management Methods for Regular Treatment of China-Gates Foundation TB Prevention and Treatment Project” is to be written after the project for better publication and dissemination both at home and abroad.

**Appendices**

1. Patient information sheets

2. Consent forms

3. Patient questionnaire at the enrollment

4. Patient management record

**Participant information sheet: control arm**

**COMMUNITY TRIAL OF NEW METHODS IN TUBERCULOSIS TREATMENT MANAGEMENT**

**Health education**

1. Tuberculosis is curable under standard treatment management with more than 6 months’ treatment.
2. Taking medicine regularly is very important for cure the disease. Due to irregular medication or interruption of the course, drug-resistant TB is developed among a number of patients.
3. The symptoms of TB will be disappeared after being treated with anti-TB drugs. However, before conversion to the sputum smear negative, patients would not speak aloud, cough, and sneeze among the public.

**Brief introduction of the new treatment management**

1. DOT require patients to go to village clinic for taking TB drugs every two days, but it would cost more and result in inconvenience for doctors and patients.
2. Now, we use an electronic medication box to assist patients’ treatment management.
3. Electronic medication box is FREE for patients who are enrolled in the study.
4. The electronic medication box we provided is only used for containing TB drugs. Please keep the box from water, children and fire.
5. Patients will return to the TB dispensary for monthly re-fills and for when to give spit specimens at 2, 5 and 6 months following start of TB treatment.
6. TB patients in other districts/counties are receiving other methods to improve TB adherence. We will then compare treatment adherence amongst patients from districts/counties receiving the other methods to improve TB adherence with patients receiving standard of care.

**INFORMED CONSENT FOR COMMUNITY TRIAL OF NEW METHOD IN TUBERCULOSIS STANDARD TREATMENT MANAGEMENT**

**(CONTROL ARM)**

Dear patients,

Tuberculosis is curable under standard treatment management. However, due to irregular medication or interruption of the course, drug-resistant TB is developed among a number of patients. This makes their condition worse and results in the occurrence of incurable tuberculosis. To ensure medication is taken timely and treatment adherence is good, National Center for Tuberculosis Control and Prevention, China CDC, is conducting a community trial of new methods in tuberculosis treatment management

We are doing the research study and would like to invite you to take part. You will receive an electronic medication box, and your treatment management is conducted with the use of the facility. All information collected during the course of this study will be kept securely and confidentially. Your name and contact details will only be available to study staff, and will be stored electronically, completely separately from the other information.

Your decision to take part or not will not affect your health care. You can decide to stop taking part in the study at any time, without giving a reason. If you have any question on the study, you can enquire your doctor.

If you decide to take part, we will ask you to sign on a consent form.

National Center for Tuberculosis Control and Prevention, China CDC

I, ______ (insert name) have read the patient information sheet and understand my participation is voluntary and I am free to withdraw at any time, without giving any reason, without my medical care or legal rights being affected.

hereby, state that I am willing to take part in the study and accept the use of electronic medicine box in the treatment management.

Patients Signature: ________________ Date: ______________

I have accurately informed the subjects of this document to the patients/recipients. He/she has accurately read informed consent and has the opportunity to raise question. I, hereby, guarantee that he/she sign this informed consent based on the voluntary principles.

Doctors (Name of Person taking consent) Signature: ___________ Date: ______________

**Participant information sheet: cell phone arm**

**COMMUNITY TRIAL OF NEW METHODS IN TUBERCULOSIS TREATMENT MANAGEMENT**

**Health education**

1. Tuberculosis is curable under standard treatment management with more than 6 months’ treatment.
2. Taking medicine regularly is very important for cure the disease. Due to irregular medication or interruption of the course, drug-resistant TB is developed among a number of patients.
3. The symptoms of TB will be disappeared after being treated with anti-TB drugs. However, before conversion to the sputum smear negative, patients would not speak aloud, cough, and sneeze among the public.

**Brief introduction of the new treatment management**

1. DOT require patients to go to village clinic for taking TB drugs every two days, but it would cost more and result in inconvenience for doctors and patients.
2. Now, we use new method with SMS and electronic medication box to conduct patients’ treatment management.
3. Electronic medication box is FREE for patients who are enrolled in the study.
4. If patient has not a cell phone, we will provide a free one for him/her who is enrolled in the study.
5. Patients will return to the TB dispensary for monthly re-fills and for when to give spit specimens at 2, 5 and 6 months following start of TB treatment.
6. TB patients in other districts/counties are receiving standard of care or other methods to improve TB adherence. We will then compare treatment adherence amongst patients from districts/counties receiving the cell phone to improve TB adherence with patients receiving standard of care. We will also compare adherence amongst patients from districts/counties receiving the other methods with patients receiving standard of care.

**What is the treatment management of cell phone?**

1. To send SMS to remind patients for taking medicine on time
   1. According to patients’ requirement, Doctor /study member will set the time when patient receives the SMS by cell phone. SMS text is “please take the medication on time” and is the same for each reminder. Once receiving the SMS of reminding, patients should take medicines and make a reply with cell phone.
   2. Up to three SMS reminders are sent to the patient on the day of medication, depending on whether the patient replies or not. These reminders are sent at the agreed time medication is to be taken and subsequently at 12noon and 6pm if no reply is received.
   3. The patient is expected to reply by SMS with or without text. Once a reply has been received the reminders stop for that day.
2. To send SMS to remind patients of the monthly dispensing visit

SMS reminder sent 4,3,2,1 days before the scheduled monthly follow-up visit.

1. Other issues of using cell phone for treatment management
   - 1. To keep cell phone working, avoid cell phone out of power, recharge fee timely;
     2. If taking medication in advanced, patient still need to reply the SMS by cell phone;
     3. If any question and problem with cell phone, please contact your doctor or study member ASAP.
     4. We will provide an electronic medication box which is only used for containing TB drugs. Please keep the box from water, children and fire.

**Participant information sheet: electronic medication box arm**

**INFORMED CONSENT FOR COMMUNITY TRIAL OF NEW METHOD IN TUBERCULOSIS STANDARD TREATMENT MANAGEMENT**

**(MOBILE PONHE ARM)**

Dear patients,

Tuberculosis is curable under standard treatment management. However, due to irregular medication or interruption of the course, drug-resistant TB is developed among a number of patients. This makes their condition worse and results in the occurrence of incurable tuberculosis. To ensure medication is taken timely and treatment adherence is good, National Center for Tuberculosis Control and Prevention, China CDC, is conducting a community trial of new methods in tuberculosis treatment management

We are doing the research study and would like to invite you to take part. You will receive a cell phone and an electronic medication box, and your treatment management is conducted with the use of the facilities. All information collected during the course of this study will be kept securely and confidentially. Your name and contact details will only be available to study staff, and will be stored electronically, completely separately from the other information.

Your decision to take part or not will not affect your health care. You can decide to stop taking part in the study at any time, without giving a reason. If you have any question on the study, you can enquire your doctor.

If you decide to take part, we will ask you to sign on a consent form.

National Center for Tuberculosis Control and Prevention, China CDC

I, ______ (insert name) have read the patient information sheet and understand my participation is voluntary and I am free to withdraw at any time, without giving any reason, without my medical care or legal rights being affected.

hereby, state that I am willing to take part in the study and accept the use of electronic medicine box in the treatment management.

Patients Signature: ________________ Date: ______________

I have accurately informed the subjects of this document to the patients/recipients. He/she has accurately read informed consent and has the opportunity to raise question. I, hereby, guarantee that he/she sign this informed consent based on the voluntary principles.

Doctors (Name of Person taking consent) Signature: _____________ Date: _______________

**COMMUNITY TRIAL OF NEW METHODS IN TUBERCULOSIS TREATMENT MANAGEMENT**

**Health education**

1. Tuberculosis is curable under standard treatment management with more than 6 months’ treatment.
2. Taking medicine regularly is very important for cure the disease. Due to irregular medication or interruption of the course, drug-resistant TB is developed among a number of patients.
3. The symptoms of TB will be disappeared after being treated with anti-TB drugs. However, before conversion to the sputum smear negative, patients would not speak aloud, cough, and sneeze among the public.

**Brief introduction of the new treatment management**

1. DOT require patients to go to village clinic for taking TB drugs every two days, but it would cost more and result in inconvenience for doctors and patients.
2. Now, we use new method with an electronic medication box to conduct patients’ treatment management.
3. Electronic medication box is **FREE** for patients who are enrolled in the study.
4. Patients will return to the TB dispensary for monthly re-fills and for when to give spit specimens at 2, 5 and 6 months following start of TB treatment.
5. TB patients in other districts/counties are receiving standard of care or other methods to improve TB adherence. We will then compare treatment adherence amongst patients from districts/counties receiving the electronic medication box to improve TB adherence with patients receiving standard of care. We will also compare adherence amongst patients from districts/counties receiving the other methods with patients receiving standard of care.

**What is the treatment management of electronic medication box?**

1. To remind patients for taking medicine with human voice or music
2. According to patients’ requirement, Doctor /study member will set an agreed time when the box remind patient to take medication in first time.
3. If the box is not opened at that time there is up to eight further reminders (bleep), taking place at 5, 20, 30 minutes, 1 hour, 2 hour, 4 hour, 6 hour and 8 hour after 30 minutes of the agreed time if the medication box is not opened.
4. Patients should open the box and take medication when receiving reminder at once. Once the box has been opened the reminders stop for that day.
5. The box will ask patient to close the box with human voice of “close the box” if the box keeps open beyond 15 seconds.
6. To remind patients of the monthly dispensing visit

Medication box reminder (human voice) 4,3,2,1 days before the scheduled monthly follow-up visit.

1. Other issues of using electronic box for treatment management
2. To keep the box working, avoid it out of power;
3. To keep the box from water, children and fire;
4. Do not open the box frequently, especially in non-taking medication day.
5. If opening the box in advanced, patient still need to reply the SMS by cell phone;
6. If any question and problem with the box happened, please contact your doctor or study member ASAP.

**INFORMED CONSENT FOR COMMUNITY TRIAL OF NEW METHOD IN TUBERCULOSIS STANDARD TREATMENT MANAGEMENT**

**(MEDICATION MONITOR ARM)**

Dear patients,

Tuberculosis is curable under standard treatment management. However, due to irregular medication or interruption of the course, drug-resistant TB is developed among a number of patients. This makes their condition worse and results in the occurrence of incurable tuberculosis. To ensure medication is taken timely and treatment adherence is good, National Center for Tuberculosis Control and Prevention, China CDC, is conducting a community trial of new methods in tuberculosis treatment management

We are doing the research study and would like to invite you to take part. You will receive an electronic medication box, and your treatment management is conducted with the use of the facility. All information collected during the course of this study will be kept securely and confidentially. Your name and contact details will only be available to study staff, and will be stored electronically, completely separately from the other information.

Your decision to take part or not will not affect your health care. You can decide to stop taking part in the study at any time, without giving a reason. If you have any question on the study, you can enquire your doctor.

If you decide to take part, we will ask you to sign on a consent form.

National Center for Tuberculosis Control and Prevention, China CDC

I, ______ (insert name) have read the patient information sheet and understand my participation is voluntary and I am free to withdraw at any time, without giving any reason, without my medical care or legal rights being affected.

hereby, state that I am willing to take part in the study and accept the use of electronic medicine box in the treatment management.

Patients Signature: ________________ Date: ______________

I have accurately informed the subjects of this document to the patients/recipients. He/she has accurately read informed consent and has the opportunity to raise question. I, hereby, guarantee that he/she sign this informed consent based on the voluntary principles.

Doctors (Name of Person taking consent) Signature: ___________ Date: ______________

**Participant information sheet: electronic medication box and cell phone arm**

**COMMUNITY TRIAL OF NEW METHODS IN TUBERCULOSIS TREATMENT MANAGEMENT**

Health education

1. Tuberculosis is curable under standard treatment management with more than 6 months’ treatment.
2. Taking medicine regularly is very important for cure the disease. Due to irregular medication or interruption of the course, drug-resistant TB is developed among a number of patients.
3. The symptoms of TB will be disappeared after being treated with anti-TB drugs. However, before conversion to the sputum smear negative, patients would not speak aloud, cough, and sneeze among the public.

**Brief introduction of the new treatment management**

1. DOT require patients to go to village clinic for taking TB drugs every two days, but it would cost more and result in inconvenience for doctors and patients.
2. Now, we use new method with SMS and electronic medication box to conduct patients’ treatment management.
3. Electronic medication box is FREE for patients who are enrolled in the study.
4. If patient has not a cell phone, we will provide a free one for him/her who is enrolled in the study.
5. Patients will return to the TB dispensary for monthly re-fills and for when to give spit specimens at 2, 5 and 6 months following start of TB treatment.
6. TB patients in other districts/counties are receiving standard of care or other methods to improve TB adherence. We will then compare treatment adherence amongst patients from districts/counties receiving the cell phone and the electronic medication box to improve TB adherence with patients receiving standard of care. We will also compare adherence amongst patients from districts/counties receiving the other methods with patients receiving standard of care.

**What is the treatment management of cell phone?**

1. To send SMS to remind patients for taking medicine on time
   1. According to patients’ requirement, Doctor /study member will set the time when patient receives the SMS by cell phone. SMS text is “please take the medication on time” and is the same for each reminder. Once receiving the SMS of reminding, patients should take medicines and make a reply with cell phone.
   2. Up to three SMS reminders are sent to the patient on the day of medication, depending on whether the patient replies or not. These reminders are sent at the agreed time medication is to be taken and subsequently at 12noon and 6pm if no reply is received.
   3. The patient is expected to reply by SMS with or without text. Once a reply has been received the reminders stop for that day.
2. To send SMS to remind patients of the monthly dispensing visit

SMS reminder sent 4,3,2,1 days before the scheduled monthly follow-up visit.

1. Other issues of using cell phone for treatment management
   - 1. To keep cell phone working, avoid cell phone out of power, recharge fee timely;
     2. If taking medication in advanced, patient still need to reply the SMS by cell phone;
     3. If any question and problem with cell phone, please contact your doctor or study member ASAP.

**What is the treatment management of electronic medication box?**

1. To remind patients for taking medicine with human voice or music
2. The first time of electronic box reminding patients is after 30 minutes of an agreed time of receiving first SMS.
3. If the box is not opened at that time there is up to eight further reminders (bleep), taking place at 5, 20, 30 minutes, 1 hour, 2 hour, 4 hour, 6 hour and 8 hour after 30 minutes of the agreed time if the medication box is not opened.
4. Patients should open the box and take medication when receiving reminder at once. Once the box has been opened the reminders stop for that day.
5. The box will ask patient to close the box with human voice of “close the box” if the box keeps open beyond 15 seconds.
6. To remind patients of the monthly dispensing visit

Medication box reminder (human voice) 4,3,2,1 days before the scheduled monthly follow-up visit.

1. Other issues of using electronic box for treatment management
2. To keep the box working, avoid it out of power;
3. To keep the box from water, children and fire;
4. Do not open the box frequently, especially in non-taking medication day.
5. If opening the box in advanced, patient still need to reply the SMS by cell phone;
6. If any question and problem with the box happened, please contact your doctor or study member ASAP.

**INFORMED CONSENT FOR COMMUNITY TRIAL OF NEW METHOD IN TUBERCULOSIS STANDARD TREATMENT MANAGEMENT**

**(MOBILE PONHE+ MEDICATION MONITOR ARM)**

Dear patients,

Tuberculosis is curable under standard treatment management. However, due to irregular medication or interruption of the course, drug-resistant TB is developed among a number of patients. This makes their condition worse and results in the occurrence of incurable tuberculosis. To ensure medication is taken timely and treatment adherence is good, National Center for Tuberculosis Control and Prevention, China CDC, is conducting a community trial of new methods in tuberculosis treatment management

We are doing the research study and would like to invite you to take part. You will receive a cell phone and an electronic medication box, and your treatment management is conducted with the use of the facilities. All information collected during the course of this study will be kept securely and confidentially. Your name and contact details will only be available to study staff, and will be stored electronically, completely separately from the other information.

Your decision to take part or not will not affect your health care. You can decide to stop taking part in the study at any time, without giving a reason. If you have any question on the study, you can enquire your doctor.

If you decide to take part, we will ask you to sign on a consent form.

National Center for Tuberculosis Control and Prevention, China CDC

I, ______ (insert name) have read the patient information sheet and understand my participation is voluntary and I am free to withdraw at any time, without giving any reason, without my medical care or legal rights being affected.

hereby, state that I am willing to take part in the study and accept the use of electronic medicine box in the treatment management.

Patients Signature: ________________ Date: ______________

I have accurately informed the subjects of this document to the patients/recipients. He/she has accurately read informed consent and has the opportunity to raise question. I, hereby, guarantee that he/she sign this informed consent based on the voluntary principles.

Doctors (Name of Person taking consent) Signature: _____________ Date: _______________

Annex 3

TB Patient Questionnaire

| I social-demographic information | | | | | | | | | |
| --- | --- | --- | --- | --- | --- | --- | --- | --- | --- |
| Name |  | | Mobile Phone | |  | | | | |
| Registration Date (interview date) | \|_\|_\|_\|_\|/\|_\|_\|/\|_\|_\| | | TB Patient’sRegistration Number | | \|_\|_\|_\|_\|_\|_\| | | | | |
| Gender | □_1_Male □_2_ Female | | Birth Date (Year, Month, Day) | | \|_\|_\|_\|_\|/\|_\|_\|/\|_\|_\| | | | | |
| Occupation | □_1_Students(college, high school)□_2_ Teacher □_3_Child Care Workers/Housekeepers/Waiter□_4_ Retail trade□_5_ Doctor/Nurse□_6_Worker □_7_Migrant Worker □_8_Farmer □_9_Hunter□_10_Fisherman□_11_Government Official□_12_ Retirees□_13_Unemployed/Houseworker □_14_Others □_15_ Unknown | | | | | | | | |
| Ethnicity | □_1_Han□_2_Zhuang□_3_Man□_4_Hui□_5_Miao□_6_Vaguer□_7_Tujia  □_8_Yi □_9_Mongolian □_10_ Others | | | | | | | | |
| Education Level | □_1_Illiterated □_2_ Lower Middle School □_3_ Upper Middle School(Technical School) □_4_ University or More | | | | | | | | |
| Married Status | □_1_Unmarried □_2_ First Marriage with Spouse □_3_Remarried with Spouse □_4_ Divorce □_5_ Widowed | | | | | | | | |
| Normal place of residence | □_1_ Living in household registration place □_2_Living in a place other than household registration place | | | | | | | | |
| Number of Household Members |  | | Total Household Income in Last Calendar Year | | |  | | | |
| Household Address |  | | | | | | | | |
| Distance from Home to Local TB Clinic （KM） |  | Distance from Home to Supervision Facility(KM) | |  | | | Time Cost from Home to Supervison Facility(Min) | |  |
| Medical Insurance | □_1_New Rural Cooperative Medical System□_2_Urban Residents’Medical Insurance for Urban Residence□_3_ Medical Insurance for Urban Workers  □_4_ Government employee Medical Service □_5_Commerical Insurance □_6_No Insurance | | | | | | | | |
| Smear test | □_1_Positive □_2_Negative □_3_ No test | | | | | | | | |
| II Screening Questionnaire | | | | | | | | | |
| Enrolled into Groups | □_1_ Yes □_2_ No (go to “causes not be included”) | | | | | | | | |
| Are you more than 18 years old? | □_1_Yes □_2_ No | | | | | | | | |
| Are you no mental confusion? | □_1_Yes □_2_ No | | | | | | | | |
| Note: TB patients who answers “NO” to any above three questions will not be enrolled in the study. And the survey is finished now. | | | | | | | | | |
| Do you have mental disability ? | □_1_Yes □_2_ No | | | | | | | | |
| Are you prisoner? | □_1_Yes □_2_ No | | | | | | | | |
| Do you have visual/hearing/speech disability? | □_1_Yes □_2_ No | | | | | | | | |
| Do you unable to or unwilling to be treated? | □_1_Yes (Causes: □_1_ weight less than 50kg □_2_ serious complication □_3_ individual unwillingness □_4_ others, please clarify____________ )  □_2_ No | | | | | | | | |
| Note：TB patients who answer “YES” to any above four questions will not be enrolled in the study. And the survey is finished now. | | | | | | | | | |
| Reading SMS | Q1: are you able to read SMS? □_1_Yes(if yes, please jump to question “do you agree to be enrolled” ) □_2_ No  Q2 Could your relatives read SMS? □_1_Yes □_2_ No(if no, the survey is finished and the patient will not be enrolled.)  Q3 Is the relative able to stay with you during the study period?  □_1_Yes □_2_ No(if no, the survey is finished and the patient will not be enrolled.)  Q4 Is the relative willing to remind you to take pills?  □_1_Yes □_2_ No(if no, the survey is finished and the patient will not be enrolled.) | | | | | | | | |
| Do you agree to be enrolled? | □_1_Yes □_2_ No (if no, please clarify the reason_______________) | | | | | | | | |
| Please sign in commitment and treatment files when the patient was enrolled in the study. | | | | | | | | | |
| III Enrollment Form | | | | | | | | | |
| Which kind of group is involved | □_1_  Control □_2_ Mobile phone □_3_ Medication Monitor □_4_Mobilephone-Medication Monitor | | | | | | | | |
| Study Number/ID | \|_\|_\|_\|_\|_\|_\|_\| | | | | | | | | |
| Date of enrollment (year, month, date) | \|_\|_\|_\|_\|/\|_\|_\|/\|_\|_\| | | | | | | | | |
| Training time | □_1_ less than 20min □_2_20-30min □_3_30-40min □_4_more than 40min | | | | | | | | |
| Result of training | □_1_ use MM/mobilephone by his/herself □_2_ use MM/mobilephone with help of his/her family members  □_3_did not know to use after training □_4_ refuse to use □_5_ Others（Please Clarify） | | | | | | | | |
| Mobile phone Operator | □_1_ Him/herself □_2_ With Help from Other Persons  □_3_ Not applicable | | Medication Monitor Operator | | | | | □_1_ Him/her self  □_2_ With Help from Other Persons  □_3_  Not applicable | |
| Which pattern is the patient be managed? | □_1_  Control □_2_ Mobile phone □_3_ Medication Monitor □_4_Mobilephone-Medication Monitor | | | | | | | | |

Annex 4

Cluster Randomized Trial Protocol of using Mobile Text Messaging and Medication Monitor in TB case management

**(Mobile Phone/Medication Monitor/Mobil Phone-MM/Control)**

**Patient Management Record**

Name：□

Telephone：□

Date(day/month/year)： __________________________

**Before filling in the form, please read the following instructions carefully**

**Instructions for filling out patient records management**

**1．Please use blue / black pen to fill.**

**2．Properly modified, eg：34.2 , revised to 32.4 WLM 20100915。**

**3. Selected options should be marked in ☑🗊.**

**4. The firms should be strictly filled in according to rules of program to ensure the accuracy and completeness of these firms.**

**M- Monitor / Mobile phone of provision Record**

| M- Monitor of provision | | | Mobile phone of provision | | | | |
| --- | --- | --- | --- | --- | --- | --- | --- |
| ID of M- Monitor | Date of provision | Note | Use patient’s own mobile phone | Date of provision | ID of Mobile phone | Mobile phone Number | Note |
|  |  |  | □_1_ yes  □_2_ no |  |  |  |  |

**Medication/Smearing Record**

| Monthly | Date of Medication | Dosage(Plate Number) | Date of Follow-up | Date of Smearing | Results from Smearing | |
| --- | --- | --- | --- | --- | --- | --- |
|  |  |  |  |  | Positive | Negative |
| 1 |  |  |  |  |  |  |
| 2 |  |  |  |  | □ | □ |
| 3 |  |  |  |  | □ | □ |
| 4 |  |  |  |  |  |  |
| 5 |  |  |  |  | □ | □ |
| 6 |  |  |  |  | □ | □ |

**Medication Management Recode**

| Monthly | Date of Starting  Medication | Date of follow-up | *^a^ Number that should be taken | Number of Missed Medication | | | The other record | | | *^f^ Intense Management | *^g^Date  of Transferring | *^h^ DOT | *^i^Date  of Transferring |
| --- | --- | --- | --- | --- | --- | --- | --- | --- | --- | --- | --- | --- | --- |
|  |  |  |  | *^b^ Miss Doses Recode from Mobile phone software/MM/treatment card (1) | *^c^number of empty plate while traveling  (2) | *^d^The total result of miss doses  (3)=(1)-(2) | *^e^ Record  from Mobile  phone Software | *^e^Record from Patients Description | Number  of Remaining Plates |  |  |  |  |
| 1 |  |  |  |  |  |  |  |  |  | □_1_ Yes |  | □_1_ Yes |  |
|  |  |  |  |  |  |  |  |  |  | □_2_ No |  | □_2_ No |  |
| 2 |  |  |  |  |  |  |  |  |  | □_1_ Yes |  | □_1_ Yes |  |
|  |  |  |  |  |  |  |  |  |  | □_2_ No |  | □_2_ No |  |
| 3 |  |  |  |  |  |  |  |  |  | □_1_ Yes |  | □_1_ Yes |  |
|  |  |  |  |  |  |  |  |  |  | □_2_ No |  | □_2_ No |  |
| 4 |  |  |  |  |  |  |  |  |  | □_1_ Yes |  | □_1_ Yes |  |
|  |  |  |  |  |  |  |  |  |  | □_2_ No |  | □_2_ No |  |
| 5 |  |  |  |  |  |  |  |  |  | □_1_ Yes |  | □_1_ Yes |  |
|  |  |  |  |  |  |  |  |  |  | □_2_ No |  | □_2_ No |  |
| 6 |  |  |  |  |  |  |  |  |  | □_1_ Yes |  | □_1_ Yes |  |
|  |  |  |  |  |  |  |  |  |  | □_2_ No |  | □_2_ No |  |
|  |  |  |  |  |  |  |  |  |  | □_2_ No |  | □_2_ No |  |

Note:

*^a^ # that should be taken= # days during beginning of drug taken date to visit date /2,

*^b^  # missed doses from mobile phone software: # is obtained from mobile phone software for mobile phone arm.

# missed doses from MM: # is obtained from MM for MM arm or mobile-MM arm

# missed doses from treatment card is obtained from treatment form for control arm

*^c^ # of empty plate during traveling out: # is obtained from firm of agreement of doctor.

*^d^ # total result of missed doses= # missed doses from mobile phone software/MM/ treatment card (1)- # of empty plate from firm of agreement of doctor (2).

*^e^ Record from Mobile phone Software=# is obtained from mobile phone software only for mobile phone-MM arm

*^h-I^ : these items could not been filled in for control arm.

**Side Effect and Inpatient Record**

| **Side Effect** | | | | | | | | | |
| --- | --- | --- | --- | --- | --- | --- | --- | --- | --- |
| **Side Effect**：**□_1_** Yes  **□_2_** No | | | | | | | | | |
| Rank | | Date | Stop Medication Because of Side Effect | | Date of Stopping Medication | | Retrieving Medication | Date of Retrieving Medication | |
| 1 | |  | □_1_ Yes  □_2_ No | |  | | □_1_ Yes  □_2_ No |  | |
| 2 | |  | □_1_ Yes  □_2_ No | |  | | □_1_ Yes  □_2_ No |  | |
| 3 | |  | □_1_ Yes  □_2_ No | |  | | □_1_ Yes  □_2_ No |  | |
| 4 | |  | □_1_ Yes  □_2_ No | |  | | □_1_ Yes  □_2_ No |  | |
| 5 | |  | □_1_ Yes  □_2_ No | |  | | □_1_ Yes  □_2_ No |  | |
| 6 | |  | □_1_ Yes  □_2_ No | |  | | □_1_ Yes  □_2_ No |  | |
| **Inpatient** | | | | | | | | |  |
| **Inpatient：□_1_** Yes  **□_2_** No | | | | | | | | |  |
| Rank | Inpatient Date | | | Discharge Date | | Stopping Medication | | |  |
| 1 |  | | |  | | □_1_ Yes  □_2_ No | | |  |
| 2 |  | | |  | | □_1_ Yes  □_2_ No | | |  |
| 3 |  | | |  | | □_1_ Yes  □_2_ No | | |  |
| 4 |  | | |  | | □_1_ Yes  □_2_ No | | |  |
| 5 |  | | |  | | □_1_ Yes  □_2_ No | | |  |
| 6 |  | | |  | | □_1_ Yes  □_2_ No | | |  |

| **Medication Monitor Usage Record** | | | | |
| --- | --- | --- | --- | --- |
| M- Monitor Failure | | □_1_ Yes  □_2_ No | | |
| Rank | Date | Reason | Resolved | Method |
| 1 |  | □_1_ Set incorrectly by doctor  □_2_ Used incorrectly by patient  □_3_ M- Monitor Failure  □_4_ No power  □_5_ Others（note） | □_1_ Yes  □_2_ No | □_1_ Retraining  □_2_ Change the M- Monitor  （the number of new M- Monitor： ）  □_3_ change the power  □_4_ Others（please Clarify） |
| 2 |  | □_1_ Set incorrectly by doctor  □_2_ Used incorrectly by patient  □_3_ M- Monitor Failure  □_4_ No power  □_5_ Others（please clarify） | □_1_ Yes  □_2_ No | □_1_ Retraining  □_2_ Change the M- Monitor  （the number of new M- Monitor： ）  □_3_ change the power  □_4_ Others（please Clarify） |
| 3 |  | □_1_ Set incorrectly by doctor  □_2_ Used incorrectly by patient  □_3_ M- Monitor Failure  □_4_ No power  □_5_ Others（please clarify） | □_1_ Yes  □_2_ No | □_1_ Retraining  □_2_ Change the M- Monitor  （the number of new M- Monitor： ）  □_3_ change the power  □_4_ Others（please Clarify） |
| 4 |  | □_1_ Set incorrectly by doctor  □_2_ Used incorrectly by patient  □_3_ M- Monitor Failure  □_4_ No power  □_5_ Others（please clarify） | □_1_ Yes  □_2_ No | □_1_ Retraining  □_2_ Change the M- Monitor  （the number of new M- Monitor： ）  □_3_ change the power  □_4_ Others（please Clarify） |
| 5 |  | □_1_ Set incorrectly by doctor  □_2_ Used incorrectly by patient  □_3_ M- Monitor Failure  □_4_ No power  □_5_ Others（please clarify） | □_1_ Yes  □_2_ No | □_1_ Retraining  □_2_ Change the M- Monitor  （the number of new M- Monitor： ）  □_3_ change the power  □_4_ Others（please Clarify） |

| **Mobile phone Usage Record** | | | | |
| --- | --- | --- | --- | --- |
| Mobile phone Failure | | □_1_ Yes  □_2_ No | | |
| Rank | Date | Reason | Resolved | Method |
| 1 |  | □_1_ Used incorrectly by patient  □_2_Net Failure  □_3_Mobile arrears  □_4_Others | □_1_ Yes  □_2_ No | □_1_ Retraining  □_2_ Recovery Network  □_3_ Recharge  □_4_ Others（note） |
| 2 |  | □_1_ Used incorrectly by patient  □_2_Net Failure  □_3_Mobile arrears  □_4_Others | □_1_ Yes  □_2_ No | □_1_ Retraining  □_2_ Recovery Network  □_3_ Recharge  □_4_ Others（note） |
| 3 |  | □_1_ Used incorrectly by patient  □_2_Net Failure  □_3_Mobile arrears  □_4_Others | □_1_ Yes  □_2_ No | □_1_ Retraining  □_2_ Recovery Network  □_3_ Recharge  □_4_ Others（note） |
| 4 |  | □_1_ Used incorrectly by patient  □_2_Net Failure  □_3_Mobile arrears  □_4_Others | □_1_ Yes  □_2_ No | □_1_ Retraining  □_2_ Recovery Network  □_3_ Recharge  □_4_ Others（note） |
| 5 |  | □_1_ Used incorrectly by patient  □_2_Net Failure  □_3_Mobile arrears  □_4_Others | □_1_ Yes  □_2_ No | □_1_ Retraining  □_2_ Recovery Network  □_3_ Recharge  □_4_ Others（note） |

**Termination of observation**

| Withdraw from Study | |
| --- | --- |
| Whether the patient offered to change the management model： | □_1_ Yes □_2_ No |
| Whether the patient will travel away more than a month： | □_1_ Yes □_2_ No |
| Date of the change： |  |
| Reason（Multiple options）： | □_1_ Can not Use Mobile  □_2_ Can not Use M- Monitor  □_3_ Mobile Failure  □_4_ M- Monitor Failure  □_5_ Complexity of model  □_6_ travel away more than a month  □_7_  Others（please Clarify） |

| **Termination** | |
| --- | --- |
| Date |  |
| Reason（Individual choice）： | □_1_ cure  □_2_ finish course  □_3_ dead(from TB/ Not from TB)  □_4_ Missed out  □_5_ Side Effect  □_6_ Diagnostic Changes ,Refused to rule, Transfer into the treatment of MDR  □_7_ Transfer out (treatment outcome _________)  □_7_ Others(please Clarify)： |

**Mobile phones\Medication Monitor Record**

| The Return of mobile | □_1_ Yes  □_2_ No | Date： |  |
| --- | --- | --- | --- |
| The Return of M-Monitor | □_1_ Yes  □_2_ No | Date： |  |

**Required to report information**

- Patient Questionnaire
- Medication/Smearing Record
- Medication Management Recode
- Side Effect and Inpatient Record
- M-mobile/ Mobile phone Usage Record
- Termination of the Observed
- Mobile phones\Medication Monitor Record

**Audit Statement**

- Informed consent form signed
- Verify that the names of patients, study number, home address, telephone number complete true and complete.
- Check into the patients met the protocol criteria.
- Research records of all projects completed.
- Complete information reported.
- Verify that the patients who did not complete the trial, already filled out the reason.
- The right to modify.

Signature：

Date：

**References:**

[1]Sudharshanie Bechan et al. Transactions of The Royal Society of Tropical Medicine and Hygiene，1997，91:704-707.

[2]World Health Organization. Global tuberculosis control： surveillance，planning， financing： WHO report 2008［R］. Geneva： WHO，2008.

[3]Pym ,A. S. and S. T. Co le, Po st DO TS, Po st genom ics: the next century of tuberculo sis control [J ]. L ancet, 1999, 353(9157) : 1004-1005.

[4] What is DOTS? <http://www.who.int/gtb/dots/whatisdots.htm>

[5] Dye, C. P ro spects for world wide tuberculosis control under the WHO DOT s strategy [ J ]. D irectly observed short -course therapy. L ancet, 1998. 352 (9144) : p. 1886291.

[6]卫生部疾病预防控制局，卫生部医政司，中国疾病预防控制中心. 中国结核病防治规划实施工作指南[M] . 北京:中国协和医科大学出版社,2009. 63.

[7]<Http://www.chinatb.org>

[8]<http://www.chinatb.org/pdf/07.pdf>

[9]WHO (2007) Global Tuberculosis Control: Surveillance, Planning, Financing: WHO Report. WHO ⁄HTM ⁄ TB ⁄ 2007 ⁄ 376.

[10]Broekmans J . Tuberculosis back to the future [J ] . Indian J Tuberc , 1994 , 41 : 2752278.

[11] Wilkinson D. Coping with Af rica’s increasing tuberculosis burden : are community supervisors an essential component of the DOT st rategy [J ] . Trop Med Int Health ,1997 , 2 : 70027041

[12] Paul Garner et al. BMJ，2003，327; 823-824

[13] Bastian I, Stapledon R, Colebunders R. Current thinking on the management of tuberculosis [J]. Curr Opin Pulm Med, 2003; 9( 3) :186- 192.

[14] Marrero A, Caminero JA, Rodriguez R, et al. Towards elimination of tuberculosis in a low income country: the experience of Cuba, 1962- 97[J]. Thorax, 2000; 55: 39- 45.

[15]Volmink J & Garner P (2006) Directly observed therapy for treating tuberculosis. Cochrane Database Syst Review (April 19)2, CD003343.

[16] Joimmy Volmink, Patrice Matchaba, Paul Garner. Directly observed therapy and treatment adherence[J]. Lancet, 2000; 355: 1345-1350.

[17] Volmink J, Garner P.Interventions for promoting adherence to tuberculosis management [J]. Cochrane Database of Systematic Reviews,2000;( 4) : CD000010.

[18] Volmink J, Matchaba P, Garner P. Directly observed therapy and treatment adherence[J]. Lancet, 2000; 355( 9212) : 1345- 1350.

[19]胡代玉，汪涛，刘晓云等.重庆市涂阳肺结核病人直接面视下服药的实施现状与影响因素分析[J].中国卫生资源，2006，9（5）：219-221.

[20]Moulding T, Onstad GD & Sbarbaro JA (1970) Supervision of outpatient drug therapy with the medication monitor. Annals of Internal Medicine 73, 559–564.

[21]World Health Organization. Global tuberculosis control： surveillance，planning， financing： WHO report 2008［R］. Geneva： WHO，2008.

[22]Sun Q，Meng Q，Yip W，et al． DOT in rural China：experience from a case study in Shandong Province，China［J］．Int J Tuberc Lung Dis，2008，12（6）：625－630

[23]Hu D，Liu X，Chen J，et al． Direct observation and adherence to tuberculosis treatment in Chongqing，China：a descriptivestudy［J］． Health Policy Plan，2008，23（1）：43－55

[24]程晓呤， 巩文水. 农村结核病人全程医疗管理辅以家庭督导的效果分析［J］. 海峡预防医学杂志， 2004， 10： 1.

[25]Lienhardt C & Ogden JA (2004) Tuberculosis control in resource poor countries: have we reached the limits of the universal paradigm? Tropical Medicine and International Health 9, 833–841.

[26]Rusen ID, Aid-Khalid N & Alarcon E (2007) Cochrane systematic review of directly observed therapy for treating tuberculosis –good analysis of the wrong outcome. International Journal of Tuberculosis and Lung Disease 11, 120–121.

[27]Khan MA, Walley JD, Witter SN, Imran A & Safdar N (2002) Costs and cost effectiveness of different DOT strategies for the treatment of tuberculosis in Pakistan. Health Policy Plan 17, 178–186.

[28]陈静，胡代玉，汪洋等. 对涂阳肺结核病人实施直接面视下服药影响因素定性研究[J]. 现代预防医学，2006，33(11):2024-2026.

[29]施华芳，姜冬九，李乐之等. 病人依从性的研究进展[J]. 中华护理杂志, 2003,38(2):134.

[30] Moore P． DOTS－what’s in a name ［J］ Lancet，2001，357（9260）：940

[31] Zignol M，Hosseini MS，Wright A，et al． Global incidence of multidrug-resistant tuberculosis ［J］． J Infect Dis，2006，194（15）：479－485

[32] Sun Q，Meng Q，Yip W，et al． DOT in rural China：experience from a case study in Shandong Province，China［J］．Int J Tuberc Lung Dis，2008，12（6）：625－630

[33] Hu D，Liu X，Chen J，et al． Direct observation and adherence to tuberculosis treatment in Chongqing，China：a descriptivestudy［J］． Health Policy Plan，2008，23（1）：43－55

[34] 郑玉群，王建明，周扬等. 江苏省涂阳肺结核患者治疗依从性现状调查及影响因素分析［J］.南京医科大学学报，2009，29（10）：1384.

[35] Shargie EB，Lindtjorn B． Determinants of treatment adherence among smear-positive pulmonary tuberculosis patients in Southern Ethiopia［J］． PLoS Med，2007，4（2）：e37

[36] Mateus-Solarte JC，Carvajal-Barona R． Factors predictive of adherence to tuberculosis treatment，Valle del Cauca，Colombia［J］． Int J Tuberc Lung Dis，2008，12（5）：520－526

[37] Ormerod L P , Prescott RJ . Interrelations between relapses ,drug regimens and compliance with treatment in tuberculosis[J ] . Respir Med , 1991 ,85 (3) :239.

[38 ]吕姿之. 健康教育与健康促进[M] . 北京:北京医科大学出版社,1998. 53.

[39] Tam C M, Leung C C , Noertjojo K, et al. Tuberculosis in Hong Kong patient characteristics and treatment outcome[J ] . Hong Kong Med J , 2003 , 9 (2) : 83.

[40] Thorson A , Diwan V K. Gender inequalities in tuberculosis : aspects of infection , notification rates and compliance[J ] . Curr Opin , 2001 ,7 (3) :165.

[41]黄春艳. 抗结核药致肝功损害的防治及护理[J ] . 现代护理,2002 ,8 (10) :782.

[42]徐佳薇,胡代玉,张拓红,等. 改善结核病人规则服药依从性的定性研究[J ] . 重庆医科大学学报,2007 ,32 (9) :977 - 979.

[ 43 ] 张贵祥,石寒冰. 结核病人规则服药依从性探讨[J ] . 齐齐哈尔医学院学报,2006 ,27 (2) :176 - 177.

[44]高翠南. 肺结核病人规则服药依从性的探讨[ J ] . 中国防痨杂志,2002 ,24 (5) :273 - 274.

[45]曾国艳,刘剑梅,邓晓丽,等. 健康教育提高肺结核患者遵医行为的研究[J ] . 护士进修杂志,2001 ,16 (11) :807.

[46]肖惠敏,姜小鹰,陈晓春. 高血压病人服药依从性的研究进展[J ] . 中华护理杂志,2003 ,38 (1) :46.

[47] Palanduz A , Gultekin D , Erdem E , et al. Low level of compliance with tuberculosis treatment in children : monitoring by urine tests [J ] . Ann Trop Paediatr , 2003 , 23 (1) :47.

[48] 刘贤臣. 论病人的依从性[J ] . 国外医学社会医学分册,1988 ,5 (4) :237.

[49] Tuberculosis Coalition for Technical Assistance (2006) International Standards for Tuberculosis Care (ISTC). Tuberculosis Coalition for Technical Assistance, The Hague.

[50] Claxton AJ, Cramer J, Pierce C. A systematic review of the associations between dose regimens and medication compliance. Clinical Therapeutics 2001;23(8):1296–1310.

[51] de Bruin M, Hospers HJ, van den Borne HW, Kok G, Prins JM. Theory-and evidence-based intervention to improve adherence to antiretroviral therapy among HIV-infected patients in the Netherlands: A pilot study. AIDS Patient Care and STDs 2005;19(6):384–394.

[52] Hinkin CH, Castellon SA, Durvasula RS, Hardy DJ, Lam MN, Mason KI, et al. Medication adherence among HIV+ adults: Effects of cognitive dysfunction and regimen complexity. Neurology 2002;59 (12):1944–1950.

[53] Samet JH, Sullivan LM, Traphagen ET, Ickovics JR. Measuring adherence among HIV-infected persons: Is MEMS consummate technology? AIDS and Behavior 2001;5(1):21–30.

[54]Menzies D, Dion MJ, Francis D et al. (2005) In closely monitored patients, adherence in the first month predicts completion of therapy for latent tuberculosis infection. International Journal of Tuberculosis and Lung Disease 9, 1343–1348.

[557] Moulding TS & Caymittes M (2002) Managing medication compliance of tuberculosis patients in Haiti with medication monitors. International Journal of Tuberculosis and Lung Disorders6, 313–319.

[56]蔡红琼，涂光敏，白丽琼等.电话督导对肺结核患者治疗管理的效果分析［J］.实用预防医学.2009,16(2),439-440

[57] Sonnenberg P , Ross M H ,Shearer S C ,et al . The effect of dosagecards on compliance wit h directly observed t herapy inhospital [J ] .Int J Tuberc Lung Dis ,1998 ,2 (2) :168.

[58] Paramasivan R. Short course chemotherapy : a cont rolled study of indirect defaulter ret rieval method [J ] . Indian J Tuberc , 1993 , 40 : 18521901

[59 ] Volmink J . Systematic review of randomised cont rolled trials of st rategies to promote adherence to tuberculosis treatment [J ] . BMJ , 1997 , 315 : 140321406.
