# Supplementary material for: Effectiveness of Electronic Reminders to Improve Medication Adherence in Tuberculosis Patients: A Cluster-Randomised Trial
Source: PLoS Med. 2015 Sep 15;12(9):e1001876. doi: 10.1371/journal.pmed.1001876 (PMC4570796; doi:10.1371/journal.pmed.1001876)
Supplement: S3 Text — (DOCX) [file pmed.1001876.s009.docx]

**S3 Text. Trial methods and results**

***Methods:***

***Cluster selection***

Clusters were defined as counties/districts within four purposively selected regions in China (three provinces: Heilongjiang, Jiangsu, Hunan; and one direct-controlled municipality: Chongqing). For the purposes of this study, counties/districts were defined depending on whether more/less than half the population were agricultural. Within each of the four regions, six counties and three districts were selected that each registered at least 300 active pulmonary TB patients in 2009 and the county/district TB Control Program was judged as capable and willing to participate in this study. In the three provinces, five counties/districts were selected from one city and four from another city, while in Chongqing nine counties/districts were selected from across the municipality.

***Study endpoints***

The two tuberculosis treatment outcome endpoints followed standard definitions (ref WHO). The first endpoint was “poor treatment outcome”, defined as any of failure, death or patient loss to follow-up, with the denominator restricted to any of these and cure/completed treatment. The second was “patient loss to follow-up”, with the denominator restricted to patient loss to follow-up and cure/completed treatment. If a patient was transferred out to another clinic then the treatment outcome as reported by the subsequent clinic was used for the secondary outcome measurement, where possible.

***Sample size: additional calculations***

Assuming a coefficient of variation in true proportions between clusters of 0.3, 9 clusters per arm, a two-sided type I error of 0.05, the percentage with “poor adherence” in the control arm of 30%, 110 TB patients per cluster would be required to detect a 40% reduction in the outcome in the intervention arm, with power 81% .

***Randomisation***

The random allocation of the 36 clusters to the four arms was stratified by the 24/12 counties/districts (representing rural/urban stratification) and restricted such that each province had at least two clusters in each arm. To calculate the impact of this restriction, 10,000 stratified random allocations were generated, of which 33 met this restriction criteria (0.33%, 95% CI: 0.23%, 0.46%). The stratification resulted in 8x10^17^ possible allocations, which the restriction reduced to an estimated 3x10^15^ possibilities, which was judged sufficient. The probability that any two clusters would be in the same arm was determined from 5,000 randomly generated, acceptable allocations and varied from 0.12 to 0.27. One of these 5,000 acceptable allocations was then randomly chosen as the final allocation based on the runiform command in Stata version 12.0. The randomisation was conducted by the statistician (JJL).

***Analysis***

Patients who defaulted had non-adherence imputed for each month following default for all four adherence endpoints.

Analysis for all endpoints used standard methods for a small number of clusters per arm (ref Hayes and Moulton), accounting for the stratified design and giving each cluster equal weight. Endpoints were aggregated at the cluster level using the arithmetic mean for quantitative adherence endpoints and percentages for binary treatment outcome endpoints. Log transformations were applied to each cluster-level endpoint and approximate standard errors for the mean differences in log-transformed cluster-level endpoints were calculated from the residual mean square of two-way analyses of variance on arm, stratum and their interaction. Adjusted analyses were conducted using a two stage approach (ref) with the first stage using regressions to calculate predicted outcomes for each individual, based on their characteristics and stratum. Pre-specified sub-group analyses for the primary outcome were: age group; literacy; gender; and rural/urban.

There was a problem with the battery for some medication monitors, such that either the battery connection had a fault or the battery ran down very quickly, which resulted in power outages that could be easily fixed by the patient or doctor if they became aware of it. Data collected prior to a power outage were not lost. However, if the medication monitor was opened during a power outage, the date and time of opening was not captured, potentially leading to an over-estimate of non-adherence. If the power outage was resolved before the next patient-doctor appointment, any subsequent dates were captured incorrectly, because the internal clock reset to a date in 2010. If the power outage was resolved at a patient-doctor appointment, the doctor could reset the internal clock to the correct date, ensuring that subsequent dates were collected correctly (unless another power outage occurred). Since any incorrectly captured dates were easily identifiable, it was possible to conduct a sensitivity analysis separating patients into those who had any report of a medication monitor problem, or had incorrect dates recorded by the medication monitor, versus those who did not.

***Results:***

Of the 1223 TB patients who were ineligible for the study, the most common reason was being unable to use the mobile phone to SMS (66% overall [732/1146], ranging from 55% to 73% across arms), followed by having a communication disability (17% overall [191/1446], ranging from 12% to 22% across arms) and being <18 years of age (14% overall [162/1446], ranging from 11% to 18% across arms).

Secondary outcome of smear conversion at 2 month: The data on smear conversion were sparse. Of the 4173 TB patients in the study only 36% (n=1513) were smear positive at baseline and of these n=1346 (89%) have a 2-month smear result recorded. Overall 3.6% (49/1346) were smear-positive at 2 months; by arm the results were as follows – control 2.1% (7/332), text messaging 5.6% (19/337), medication monitor 3.1% (11/356), combined 3.7% (12/321). No formal comparisons by study arm were performed due to small number of outcomes.
